# Digital Data Donation With Adolescents

**DOI:** 10.1111/nyas.70140

**Published:** 2025-11-10

**Authors:** Valerie Z. Y. Yap, Amira Skeggs, Amanda M. Ferguson, Amelia Leyland‐Craggs, Laura Boeschoten, Kasper Welbers, Sebastian Kurten, Amy Orben

**Affiliations:** ^1^ Medical Research Council Cognition and Brain Sciences Unit University of Cambridge Cambridge United Kingdom; ^2^ Department of Methodology and Statistics Utrecht University Utrecht the Netherlands; ^3^ Department of Communication Science Vrije Universiteit Amsterdam Amsterdam Netherlands; ^4^ Department of Interdisciplinary Social Science Utrecht University Utrecht Netherlands

**Keywords:** adolescents, data collection, data donation, data infrastructure, digital media, social media

## Abstract

Growing concerns about digital media's impact on adolescent well‐being highlight critical limitations in existing research methodologies that rely predominantly on self‐reported screen time measures, which inadequately capture the complexity of digital interactions and behavioral patterns. Data donation, where individuals voluntarily share objective social media data, offers a transformative approach, yet its feasibility with adolescents remains underexplored. This study evaluated the feasibility of implementing data donation methodology with adolescent populations to develop practical guidelines for future studies. We conducted a large‐scale, 2‐week longitudinal trial (*N* = 358, aged 13–18) alongside focus groups and youth advisory panels, integrating ecological momentary assessment (EMA), validated psychometrics, and data donation from Instagram and TikTok. Results demonstrated strong feasibility across technical, ethical, and engagement dimensions: Overall, 78.9% of participants donated Instagram data, and 65.8% donated TikTok data, with 74% average EMA response rates. Findings indicate substantial willingness to participate in intensive digital behavioral research when appropriate safeguards and youth‐centered approaches are implemented. We propose six key recommendations for data donation studies with adolescents: (1) clearly communicating research value, (2) establishing rigorous consent procedures, (3) centering adolescents’ voices through co‑design, (4) selecting appropriate platforms, (5) implementing suitable technical frameworks, and (6) building robust multi‑stakeholder recruitment strategies.

## 1 Introduction

Digital media has become an integral part of childhood, with nearly 99% of children in the United Kingdom spending time online [1]. As children transition into adolescence, their exposure to digital environments increases significantly, with 9 in 10 British children gaining access to their own mobile phones by the age of 11 [1]. By age 16, they estimate spending an average of 4 h and 54 min online daily [2]. As adolescents undergo substantial neurobiological, behavioral, and environmental development, some fear this will interact with the social and interactive nature of popular digital media, such as social media, to increase vulnerability to mental health problems [3]. Considering the significant amount of time adolescents spend online, accurately measuring their activities on digital platforms is therefore crucial.

Current research on adolescent smartphone and social media usage overwhelmingly relies on self‐report methods, which have significant limitations in accurately capturing the complexity and scope of these behaviors [4, 5]. One of the most popular self‐report approaches asks participants to estimate the amount of time spent on or the frequency of their smartphone or social media use over a given period [6]. This method, however, fails to account for diverse types of online interactions or the potential multitasking nature of online activities, leading to unreliable data [7]. Further, the accuracy of self‐reports diminishes over longer reference periods, especially for daily, habitual behaviors like smartphone use, as individuals struggle to recall mundane actions [8]. Young users, in particular, face greater challenges in accurately estimating their time spent online due to the frequency and intensity of their engagement [9]. Social desirability bias complicates these reports further, as excessive smartphone and social media use are often viewed negatively, prompting users to potentially underreport their usage [10].

To combat the limitations of self‐reports of time spent on smartphones or social media, inbuilt applications and tools have now been developed to accurately track time spent on applications or screens (e.g., Apple Screen Time). However, although used for research, these tools are often black‐boxes and do not provide the nuanced data needed for high‐quality public health research [11], particularly as they are limited to measures of time spent and therefore cannot help us understand the effects of, for example, different types of digital environments or contents [12]. Other research projects have therefore turned to apps and software to monitor activities or content users engage in on various applications or devices. For example, the Stanford Human Screenome Project [13] employs background software that captures screenshots of a volunteer's phone every 5 s while in use. However, projects like this demand considerable financial investment for development, secure data storage, and ongoing analysis. Beyond logistics, an app recording user activity for long periods of time raises ethical and participant recruitment concerns.

Better measurement alternatives are far and few between. The overreliance on self‐report methods in research on digital platforms is partly driven by technology companies’ reluctance to share detailed user data with academic researchers. Although many companies share data with other third parties (e.g., advertisers) [14], access remains complicated for researchers and varies significantly across different platforms and regions. Researchers have often been forced to rely on application programming interfaces (APIs), web scraping, and other informal methods to collect platform data due to the lack of legal mandates for data sharing. APIs are platform‐provided tools that give external parties structured access to selected types of data (e.g., post counts, likes, or follower metrics). Although they once offered a critical channel for research, platforms strictly control their scope, and in recent years many APIs have been curtailed or shut down entirely [15]. As a result, these approaches to data acquisition are becoming increasingly unreliable, leaving researchers without systematic ways to study platform effects.

To address these gaps and improve how we understand adolescent digital technology use, more robust and reliable measurement tools are needed. This is especially the case for methods that can capture the complexity of online behavior without relying solely on adolescents’ ability to accurately recall their activities and platforms’ engagement with data access schemes for independent researchers. These measurement tools should also be low‐effort, ethically vetted, and scalable to ensure easy implementation and broader participation that yields more accurate insights.

In this context, data donation refers to the voluntary transfer of social media data from adolescents to researchers. Described in detail in the next section, it emerges as a promising solution to gather nuanced social media data while empowering adolescents to take control of their online presence. However, little is known about the feasibility and acceptability of data donation for adolescent populations, who are unique in their vulnerabilities, aims, and engagement mechanisms with research. Their cognitive, emotional, and social attributes profoundly influence online behaviors and perceptions of privacy and risk [16, 17]. Unlike adults, adolescents often navigate their digital lives under varying degrees of parental guidance and are highly susceptible to peer influence, which can significantly shape their willingness to share personal data [18].

Previous large‐scale feasibility studies of data donation have explored adult perspectives on personal data donation, revealing that motivations are driven by perceived benefit for societal or public good [19]. There is, however, extremely limited understanding of young people's views of data donation, which may differ significantly from adults’ and influence their willingness and ability to participate. Furthermore, despite often being considered “digital natives,” adolescents may possess a less sophisticated understanding of data governance mechanisms and the long‐term implications of data sharing compared to older individuals [20].

Emerging research has begun to address these ethical and practical considerations by developing participatory frameworks for youth involvement in shaping data donation processes [21] and ecologically valid methods for collecting adolescent social media data [22]. However, a systematic understanding of adolescents’ own perspectives on data donation, such as their motivations, perceived risks, ethical concerns, and potential barriers, remains incomplete.

To fill this research gap, this article summarizes a feasibility trial of data donation methodology with adolescent populations, evaluating its technical functionality, participant acceptability, ethical safeguards, and scalable deployment protocols. We additionally trialed ways to boost adolescent engagement and understanding of data donation research through the creation of functionalities where they can visualize their own data before donation. This work included a range of focus groups, advisory panels, the development of a pilot data collection protocol, and the implementation of a study involving over 300 adolescents, aged 13–18 years. We synthesize key findings that emerged, addressing both the potential benefits and concerns related to data donation among adolescents, which can form a framework for future research that prioritizes the voices and experiences of adolescents when using data donation. We also use our findings to develop practical recommendations and guidelines for conducting data donation studies with this important age group.

## 2 What is Data Donation?

Data donation is a data collection method in which individuals willingly share their personal data for research purposes [23]. It leverages a fundamental aspect of data protection laws in the United Kingdom and European Union, which give individuals a right to access their personal data held by digital platforms, like social media [24]. Platforms should therefore provide a digital copy of an individual's personal data in a transportable format when requested. Most platforms address this requirement by providing individuals with .zip files containing the relevant data when requested via a button or form on the platform. We refer to the requested and obtained data as a Data Download Package (DDP) [25].

DDPs provide objective, time‐stamped data about digital platform use, addressing the inaccuracies often found in self‐reported usage, particularly among adolescents. Further, the data are not restricted to time spent on the platform but include other behaviors or experiences, such as information on posts liked, shared, or commented on and temporal usage trends. This dual access to behavioral patterns and content‐level data enables more nuanced, ecologically valid research into adolescents’ digital lives, while offering opportunities to explore both between‐ and within‐person variability in digital experiences.

In Table 1, we compare data donation to other broad methods of social media data collection: questionnaires, app tracking, and industry collaboration. Unlike methods that depend on platform collaboration or capture only limited in‐app interactions, data donation offers a comprehensive, user‐centered view of online behavior. Individuals can also actively contribute personal data from multiple platforms, making it scalable, ethically transparent, and cost‐effective. However, scalability can be hindered by technical challenges such as the size of data downloads and participant burden in requesting and uploading data. Additionally, variability in platform policies and jurisdictional regulations influences the degree of control users have over what data they share.

**TABLE 1:** Comparison of different methods to collect social media data from individual participants.

Criteria	Questionnaires	App‐tracking	Industry collaboration	Data donation
**Definition**	Self‐report measure capturing participants’ subjective experiences or behaviors related to social media use	Research‐specific applications installed on participants’ devices to passively monitor digital behaviors through custom‐developed tools that actively collect data during naturalistic device use	Direct partnerships with social media platforms to access user engagement data	Participants voluntarily donating their engagement data from a social media platform, with local processing to extract research‐relevant features
**Examples**	Online/Article surveys (recall questions, daily/experience diaries)	EARS^a^ (multi‐sensor mobile sensing); Screenomics^b^ (frequent screenshots)	Meta–COS pilot^c^ (Instagram data for well‐being); other API or data‐sharing agreements	Relevant software includes PORT^d^, DataSkop^e^, OurDataHelps.org^f^, DONA^g^
**Primary data captured**	Subjective reports of frequency or time spent using social media; emotional experiences; self‐perceptions of social media use	Exact quantified behavioral traces depend on method used but can include time on platform, interactions, scrolling patterns, notifications, and screen content	Data shared can include large‐scale, precise behavioral logs: timestamps, clicks, metadata, user journeys, algorithmic exposure	Comprehensive user‐centric usage logs: messages, search history, post metadata, platform‐generated analytics. Scope varies by platform and users’ choices
**Ease of researcher administration**	High: simple to distribute and manage; minimal technical setup required	Moderate: requires selection of tracking app, ensuring participants’ compliance with app use and data transfer	Low: complex to set up fair partnership as companies normally disinclined to engage with researchers. Dependent on corporate research priorities, legal frameworks, and platform data governance policies; lengthy approval processes. Changes can occur at short notice	Moderate: requires selection of user‐friendly data donation platform and provision of clear instructions; developing local processing scripts for diverse DDP formats
**Ease of participant engagement**	High: easy and simple process	Moderate: requires download of app and internet to transfer data	Very high: requires no or minimal engagement to collect data	Low: requires multi‐step process—requesting of data and uploading to data donation platform. High digital literacy is therefore required
**Data quality and accuracy**	Low: subjective data prone to recall bias, social desirability effects, and measurement errors; limited temporal precision	Moderate: captures actual behavior but scope often limited to device‐level metrics (time spent, app switches); may miss platform‐specific features and content interactions	Very high: access to extensive objective proprietary data; including algorithmic exposure, detailed engagement metrics, and comprehensive user journey analytics; highest precision and completeness, however can be a black‐box with little researcher oversight	High: extensive objective data on platform use, but only quality depends on platform data export completeness and participant selection bias (only motivated users participate)
**Retrospective data availability**	No. Participants cannot accurately report past behavior beyond a few days	No. App‐based tracking only captures prospective behavior from installation onward	Yes. Access to extensive historical data archives maintained by platforms can be possible, for example, spanning multiple years with complete user interaction histories	Yes. Access to comprehensive historical data exports as maintained by platforms
**Real‐time tracking availability**	No. Relies on periodic self‐report	Yes. Apps can provide continuous live data while installed	Yes. Real‐time access to data streams depending on partnership terms and platform policies	No. Typically involves static data exports (even though EU regulatory changes might mandate future real‐time data portability)
**Privacy and ethical complexity**	Low: high control for participants; easily complies with ethical standards	Moderate: extensive data capture; must address legal requirements	Moderate: companies control data use; legal and ethical safeguards required. Participant consent may be indirect through platform terms	Moderate: extensive personal data sharing with enhanced participant control through local processing
**Cost‐effectiveness**	High: low cost	Low: apps often expensive to develop and maintain; requires ongoing technical support and participant management	Variable: depends on partnership agreement. It can range from free academic access to substantial licensing fees; often requires significant negotiation	Moderate: cost‐effective when leveraging existing tools. Lower than custom app development but requires technical expertise for data processing scripts
**Scalability**	High: easily scaled to large samples with few resources	Moderate: limited by app capabilities and resources to support participants	High: highly scalable but dependent on partnership constraints limited by platform capacity, legal agreements, and approval processes	Moderate: limited by app capabilities and resources to train participants. Resources needed for technical support and data processing
**User empowerment**	Moderate: relies on participant honesty without real‐time adjustments	Low: users may lack control over what is tracked	Low‐to‐moderate: typically controlled by the platform rather than individuals	Medium‐to‐high: participants explicitly choose to download and donate data; platforms and jurisdictions affect granularity^h^

^a^ EARS: Effortless Assessment of Risk States app; mobile sensing software that passively collects smartphone use data for behavioral health research.

^b^ Screenomics: Methodology capturing high‐frequency screenshots from personal devices to reconstruct digital behavior patterns and content consumption.

^c^ Meta‐COS pilot: A collaborative initiative between Meta and the Center for Open Science (COS) providing privacy‐preserving access to Instagram user data for academic research on youth well‐being.

^d^ PORT: Privacy‐first infrastructure for donating social media data to academic research; built to enhance platform transparency and data protection.

^e^ DataSkop: German data donation platform for large‐scale citizen science, enabling contributions of platform data to studies on algorithms and media consumption.

^f^ OurDataHelps.org: Qntfy‐run initiative where individuals donate data (social media, smartphones, wearables) to support suicide prevention and mental health research.

^g^ DONA: An open‐source platform for donating messaging data from WhatsApp, Facebook, and Instagram.

^h^ User empowerment for data donation varies significantly by jurisdiction. The EU's GDPR provides robust empowerment via its comprehensive “opt‐in” model and broad individual rights (e.g., access, erasure). Conversely, the United States largely operates on an “opt‐out” basis with fragmented, sector‐ and state‐specific laws, leading to inconsistent user control. This control is further modulated by technical affordances. For example, the Next platform enables selective data sharing, a feature often unavailable when sharing raw ZIP files.

## 3 Research Protocol

**FIGURE 1:**
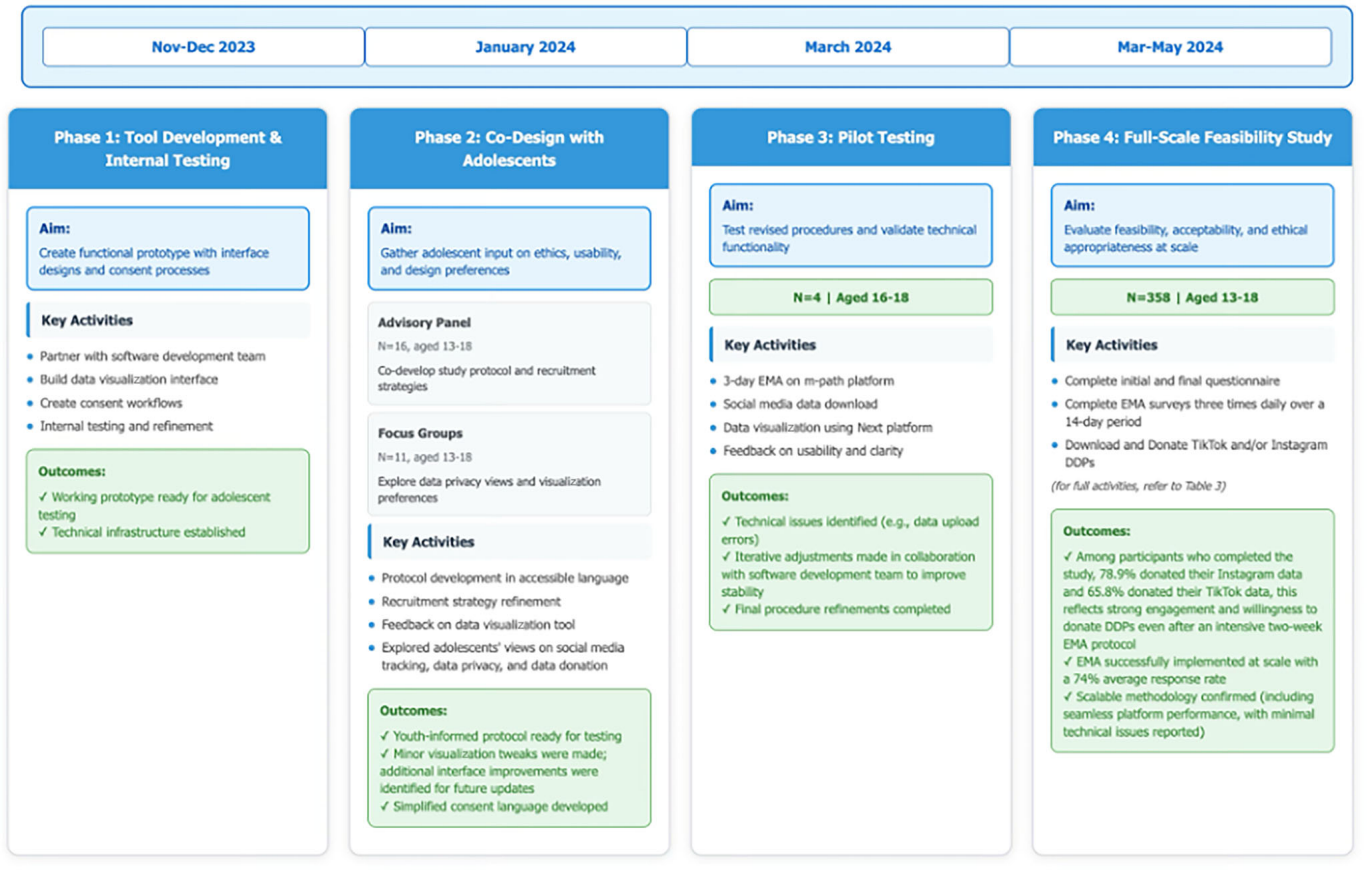
Study overview and timeline. Phase 1 (late 2023) established a working prototype ready for adolescent testing. Phase 2 (January–February 2024) was crucial for understanding whether adolescents could comprehend this novel data donation concept and gather design feedback; this reveals critical insights about visualization preferences. Phase 3 (early March 2024) specifically tested technical functionality, identifying and resolving platform issues before scale‐up. Phase 4 (March–May 2024) represented full implementation of the feasibility study and demonstrated strong engagement (78.9% Instagram, 65.8% TikTok donation rates) and successful EMA integration (74% average response rate), validating the integrated multi‐method approach for adolescent digital trace data collection. DDP, Data Download Package; EMA, ecological momentary assessment.

Our feasibility study aimed to investigate the feasibility of using data donation as a method for collecting digital social media data from adolescents (see Figure 1 for a detailed timeline of the feasibility project).

Our study also focused on creating a data visualization tool that would offer participants a way to engage with their data, understand the data they would be donating, and receive an informational benefit by engaging with the study (e.g., see how much social media they use across the week). We focused on this visualization component because (1) it has the potential to enhance transparency and informed consent by allowing participants to preview the types of data that will be shared, and (2) it makes the donation process more engaging and accessible. DDPs are typically delivered in complex and unreadable formats. By translating these files into interactive graphs and summary metrics, the visualization interface helps participants better understand their social media usage and patterns (see the Supporting Information section for mock dataset visualizations). Our study therefore trialed the feasibility and acceptability of data donation while also building this additional data visualization capability to boost both.

## 4 Advisory Panels

To ensure that our research was relevant and engaging for adolescents, we established two youth advisory panels (13–15 and 16–18 years; eight individuals each from Cambridgeshire area) to gather perspectives and inputs on data donation and the feasibility study. The panels were held in person with a familiar teacher present, along with two researchers, to create a comfortable and supportive environment. Each panel met twice for 1 h each, with 1 month between sessions.

In the first session, advisors brainstormed ideas for ensuring participant engagement in the feasibility study, focusing on how to communicate about the study in accessible language. They also provided input on potential research questions that could be addressed. In the second session, we presented the study materials and study protocol developed from the initial brainstorming session. Panel members reviewed the materials in small groups, providing feedback on areas that were too technical, simplified, or confusing. They also offered insights on recruitment strategies and potential logistical barriers to participation.

## 5 Focus Groups

We also carried out two structured focus groups to collect more in‐depth qualitative data about young people's perceptions of data donation. We interviewed a total of 11 participants (6 F, 5 M), aged 13–18 years (Group 1: 13‐ to 15‐year‐olds; Group 2: 16‐ to 18‐year‐olds). Participants identified as White (8), Asian British (2), and Slavic (1). Participants’ total household income ranged from £40,000 to over £100,000. The sessions were conducted via Zoom, each lasting 90 min, with two sessions held for each group 1 month apart. Each session was conducted by a researcher, with an additional team member moderating and taking notes. Each participant received £10/h for their time.

The focus groups also contributed to the co‐creation of a data visualization tool. This tool was built to integrate into Port, our data donation webtool (see Section 6). The Port webtool, hosted on the Next platform as a software as a service (SaaS), displays data extracted from participants’ DDPs in a user‐friendly format. After participants downloaded their DDPs from Instagram or TikTok to their local device, they visited the Next platform to view this data in a range of visualizations. The visualization tool presented key usage metrics such as video views, likes, comments, and timestamped video links as extracted from the DDPs, with personal identifiable information intentionally excluded.

The focus group input helped us to ensure that the tool was designed with adolescents’ preferences in mind. Participant feedback was gathered using Mentimeter [26] (a live polling tool) and Miro [27] (an online collaborative whiteboard) to support real‐time feedback and collaboration.

The discussions were structured around four key topics:

1. Data donation: Participants were asked about their understanding of key terms such as “personal data” and “data donation.” The discussions also explored their perspectives on donating their social media data to research, offering valuable insights into potential concerns and motivations.
2. Privacy: Participants were asked to discuss their knowledge of data privacy, their comfort around sharing data, and how they perceived the risks involved in data donation.
3. Data presentation: Participants were asked about what types of social media data they found interesting or relevant and how they would prefer to see these data presented through the data visualization tool.
4. Data visualization: To further understand participant preferences, we showed examples of graphs, charts, and tables of exemplar social media data. Participants were asked to provide feedback on which visualizations were most appealing and easy to interpret. All visualizations displayed only quantitative behavioral metrics (e.g., posting frequency, engagement counts, temporal activity patterns) that were generated from aggregated metadata (see Table 2). Importantly, no raw textual or visual content was shown, and any personally identifying information (e.g., usernames, profile images) was excluded. These visualizations aimed to inform participants of the data they were donating while upholding strong privacy standards (see the Supporting Information section for mock dataset visualizations).

**TABLE 2:** Overview of metrics and visualization approaches used in the Next platform.

Platform	Metrics displayed	Visualization approach
**TikTok**	Number of followers and following Number of likes given and received Number of videos posted Number of comments made and received Number of direct messages sent and received List of public videos viewed (with clickable links)	Summary tables presenting total counts for each activity metric (e.g., total likes given, number of videos posted) Bar graphs illustrating activity patterns over time (e.g., likes/comments/videos viewed per day or week)
**Instagram**	Number of followers and following Number of likes given and received Number of posts made and viewed Number of comments made and received Number of direct messages sent and received Number of advertisements viewed	Summary tables showing aggregate totals for each activity metric (e.g., number of comments made, number of ads viewed) Bar graphs illustrating activity patterns over time (e.g., likes/comments/videos viewed per day or week)

## 6 Technical Implementation

DDPs comprise both structured content that refers to computer‐readable data that is consistently formatted (e.g., timestamps, URLs, or numerical metrics like the number of likes or views) and unstructured content that includes open‐ended or multimedia data without a predefined format (e.g., free‐text captions, comments, story text, or images), both types of content which may contain personally identifiable information. Personally identifiable information encompasses both direct identifiers (e.g., usernames, phone numbers, email addresses, IP addresses, and profile images) and indirect or embedded identifiers (e.g., facial images, geotags, or personalized hashtags). It is very important that this is treated both safely and ethically.

To allow for safe data donation processes, our study used the Port webtool, an open‐source and highly configurable tool designed specifically to facilitate secure and privacy‐preserving data donation processes. The tool allows researchers to create configurable workflows that specify how to extract, filter, and transform relevant data from DDPs (e.g., social media activity logs) into anonymized, aggregated outputs. To enable a complete data donation process, the Port webtool is hosted on a secure web server and integrated with backend systems for storing the processed data.

In our setup, the Port webtool's frontend interface was embedded within a website powered by the Next platform, developed by the software provider Eyra. The Next platform acted as an integration hub, connecting the Port webtool with secure backend storage and other SaaS tools to facilitate a seamless and secure workflow. This is also where adolescents reviewed and visualized their social media data before deciding what to donate.

Port webtool's de‐identification pipeline systematically removes or hashes direct identifiers and filters metadata containing sensitive details like geolocation or IP addresses. Although this substantially mitigates the risk of re‐identification, unstructured content remains difficult to fully redact automatically. To mitigate this, Port performed all data processing locally on the participant's device, extracting only predefined features that are relevant to the specific research question. This ensured that researchers receive only the data necessary for analysis, whereas all other content remains securely on the participant's device.

These safeguards formed a critical component of our overall privacy framework, supplemented by our data visualization tools, options to delete data adolescents are not willing to share prior to donation, encrypted data storage, and stringent access controls to uphold confidentiality throughout the research process. For a full technical description of Port's architecture and privacy features, we refer readers to Boeschoten et al. [28], on whose work our implementation is based.

## 7 Protocol Design and Ecological Momentary Assessment (EMA)

In addition to the technical implementation of data donation and the data visualization tool, we also included an EMA component in our feasibility study (see Section 9). In the EMA component, participants are sent three surveys a day for 2 weeks to ask questions about their momentary affective states (including happiness, anxiety, and loneliness), body satisfaction, and both online and offline social interaction experiences. This design enables future studies to conduct temporally aligned analyses linking theoretically relevant psychological constructs, such as social uncertainty, body image concerns, or emotional regulation, to objective behavior metrics derived from DDPs (e.g., frequency of posting, sentiment, time of use). For example, one could examine how social uncertainty fluctuates with online behaviors, whether exposure to body‐related content on social media correlates with mood or self‐esteem shifts, or how different platforms may differentially influence well‐being.

In future studies we intend to link adolescents’ actual social media activity via DDPs with such real‐time, context‐specific data (e.g., viewed videos) and well‐being and mood; therefore, trialing this process helped us gain a more accurate understanding of the feasibility of intensive behavioral research alongside data donation.

## 8 Pilot Study

We recruited four adolescents aged 16–17 years to participate in two pilot study focus groups, held 1 week apart. The purpose of the pilot study was to test the research protocol, which included completing EMA questionnaires on the m‐path [29] platform for 3 days, downloading social media data following the instructions, and visualizing social media data using the Next [30] platform. Participants were asked to provide feedback on the clarity and length of the questionnaires, as well as the presentation and usefulness of the visualized data. The pilot was instrumental in refining study procedures, identifying potential technical issues, and ensuring the protocol could effectively support data collection for the main study.

## 9 Feasibility Study

**FIGURE 2:**
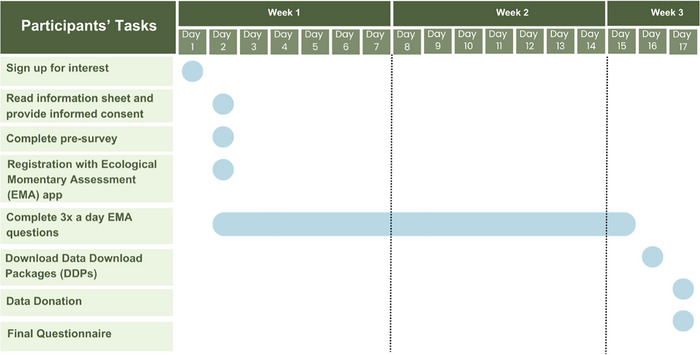
Timeline of feasibility study: This involved participants engaged in 2 weeks of EMA study, downloading social media DDPs, and giving feedback on their experiences with the data donation process.

The feasibility study aimed to assess the practicality of using data donation methods with adolescents after the extensive feedback, consultation, and design process described above (see Table 3 and Figure 2 for key steps and timelines).

**TABLE 3:** Key steps of the feasibility study.

Step	Description
**Step 1: Recruitment**	Two researchers contacted six schools to deliver presentations on social media and mental health, during which they introduced the study opportunity. Interested students were invited to sign up by scanning a QR code, which collected their contact details for follow‐up
**Step 2: Informed consent**	Participants, or their legal guardians if participants were under the age of 16, received an information sheet detailing the study's objectives and procedures. After reviewing the information, they provided informed consent for participation
**Step 3: Initial questionnaires**	Following consent, participants completed a set of demographic questions and questionnaires relating to social media use and well‐being. Specifically, this included the Strengths and Difficulties questionnaire (SDQ) [31], Online and Offline Social Sensitivity Scale (O2S3) [32], and Intolerance of Uncertainty Scale for Children (IUSC) [33] to understand social uncertainty in online relative to offline environments [34]
**Step 4: Registration with EMA study app**	Upon completing the initial questionnaires, participants were given a unique invitation code to register with the m‐path app. They downloaded the app and set it up on their devices as instructed
**Step 5: Completing the 2‐week EMA study**	For a 14‐day period, participants received three daily notifications on the m‐path app, scheduled to avoid school hours. These notifications linked to brief questionnaires that took approximately 3 min to complete, focusing on online and offline social interactions, mood, and well‐being
**Step 6: Downloading social media data (DDPs)**	At the conclusion of the 14‐day EMA phase, participants were instructed to download their social media data from TikTok and/or Instagram, following provided video and text‐based instructions. The research team was available via email for support throughout this process
**Step 7: Data donation**	After obtaining their social media data, participants uploaded the data package into the designated data donation software. They were given the option to review the data and select which components they felt comfortable donating to researcher
**Step 8: Final questionnaire**	Finally, participants were directed to complete a final set of questionnaires via Qualtrics, including follow‐up questions on social media use and mental health, data awareness and overall feedback on their study experience

A total of 558 UK‐based students signed up to the feasibility study. Of these, 358 participants provided consent to participate and completed a baseline survey (Table 4), with ages ranging from 13 to 18 years (*M* = 16.48, SD = 1.18). Participants were predominantly female, with 200 cisgender girls (55.71%) and 136 cisgender boys (37.88%). Ethnic distribution showed that most participants identified as White (*n* = 162, 45.13%), followed by Asian/Asian British (*n* = 117, 32.59%), Black (*n* = 31, 8.64%), Mixed (*n* = 17, 4.74%), and Other ethnicities (*n* = 32, 6.41%).

**TABLE 4:** Sociodemographic characteristics of participants who provided consent to the feasibility study.

Characteristic	*n*	%
**Gender**		
Cisgender boy	136	37.88
Cisgender girl	200	55.71
Non‐binary	11	3.06
Trans boy	5	1.39
Trans girls	0	0
Prefer not to say	3	0.84
Prefer to self‐describe	3	0.84
**Ethnicity**		
White	162	45.13
Asian/Asian British	117	32.59
Black/African/Caribbean	31	8.64
Mixed/Multiple ethnic	17	4.74
Other	23	6.41
Prefer not to say	9	2.51
**Age**		
13	8	2.25
14	20	5.63
15	30	8.45
16	97	27.32
17	136	38.31
18	64	18.04
**Household Income**		
Less than £20,000	33	9.71
£20,000–£39,999	67	19.72
£40,000–£59,999	31	8.66
£60,000–£99,999	46	12.85
More than £100,000	39	10.89
Not sure	118	32.96
Prefer not to say	24	6.70

*Note: N* = 358. Participants were on average 16.48 years old (SD = 1.18).

A total of 322 participants signed up for the EMA study, where they received three notifications a day to complete a short survey (<2 min) for 2 weeks. The average response rate for the EMA was 74%, with a median of 84%. At the end of the study, we received 150 Instagram DDPs and 125 TikTok DDPs through the data donation process, with 190 participants completing the final questionnaire.

## 10 Key Takeaways From Focus Groups and Advisory Panels

The engagement and completion rates in our feasibility study demonstrated participants’ willingness to share their social media data and confirmed the feasibility of this research approach. Understanding adolescents’ perspectives through the focus groups and advisory panels also helped us identify potential gaps and concerns that we might have overlooked, particularly regarding privacy, trust, and the complexity of data‐sharing processes. By engaging adolescents early in the design phase, we proactively addressed these issues and gained valuable insights into their unique viewpoints on data sharing. Given the exploratory and co‐design focus of the advisory panels and focus groups, we did not undertake a formal qualitative analysis. Key themes and insights were identified through an informal review of session transcripts, Miro board content, and researcher notes to inform study development and highlight adolescents’ perspectives on data donation. Below we cover six core takeaways about data donation with adolescents from this co‐creation process.

### 10.1 Understanding of Data Donation

Adolescents generally did not have a good understanding of data donation. Although some older adolescents (16–18 years old) had a basic understanding of data‐related concepts, their knowledge was superficial (e.g., defining data donation as “when people share their information”) and did not fully encompass the specifics of social media data. Younger adolescents (13–15 years old) displayed less awareness, with many participants being unfamiliar with the concept of data donation. Amongst both age groups, misconceptions around data donation were common, with some adolescents believing that the process involved sharing sensitive information like passwords and account details, which reflects limited awareness of safeguards built into responsible data donation workflows. Although such information may be present in raw DDPs, some data donation workflows implement local data extractors that process the DDP files and expose only predefined, research‐relevant subsets of data to participants. By filtering out sensitive content by default and requiring explicit participant consent for each data type shared, these workflows can prevent the inadvertent disclosure of personally sensitive information.

### 10.2 Attitudes Toward Data Donation

Adolescents were generally comfortable with donating their data for research purposes. Their rationale was often informed by the belief that their data was already being used by companies without their consent and feelings of resignation about their own data sovereignty. For example, participants expressed sentiments, such as “Google is collecting my data anyway without compensating, I would rather donate to a trusted source,” reflecting a sense of disillusionment about data control. Generally, participants felt more confident in donating data for research, where they were “happy to help researchers achieve positive outcomes,” especially when the goals were framed as beneficial or socially valuable.

Importantly, participants’ willingness to share data was contingent on several conditions, such as the legitimacy and trustworthiness of the research and researchers involved. Adolescents emphasized this with statements like “only for legitimate research purposes” and “make sure it is a trusted site/researcher,” highlighting the importance of credibility and transparency in gaining the trust of adolescent participants. Therefore, researchers should prioritize clear communication about their affiliations, the purpose of the study, and the intended use of the donated data to ensure their participants feel secure in their involvement.

### 10.3 Anonymous Data

Adolescents drew firm boundaries when it came to sharing personally identifiable information. They were reluctant to share data points such as full name and address. Many participants noted that they were “comfortable if the data is anonymous,” indicating that the protection of their personal identity was a key concern. This concern extended beyond their own data to include the data of others, with some participants expressing a sense of responsibility in wanting to “protect others’ data too.” This suggests that adolescents are not only aware of privacy issues but are also considerate of the implications their data sharing might have for others.

### 10.4 Social Media Data Preference

Both youth advisors and focus group participants provided insights into the types of social media data they found interesting and relevant, as well as their preferred formats for viewing this data if provided. Youth advisors, in particular, highlighted ways to make our data visualization tool more engaging for adolescents. For example, they recommended adding features that allow participants to monitor the percentage of different content types they engage with, which could also serve as a weekly reflection or accountability tool. Similarly, adolescents expressed a strong preference for organizing content by genre, especially on platforms like YouTube or TikTok, where there is a vast range of content. They suggested that categorizing their consumed content, such as entertainment, educational, or hobby‐related videos, would help them gain a clearer understanding of their viewing habits and preferences.

### 10.5 Data Visualization Preference

**FIGURE 3:**
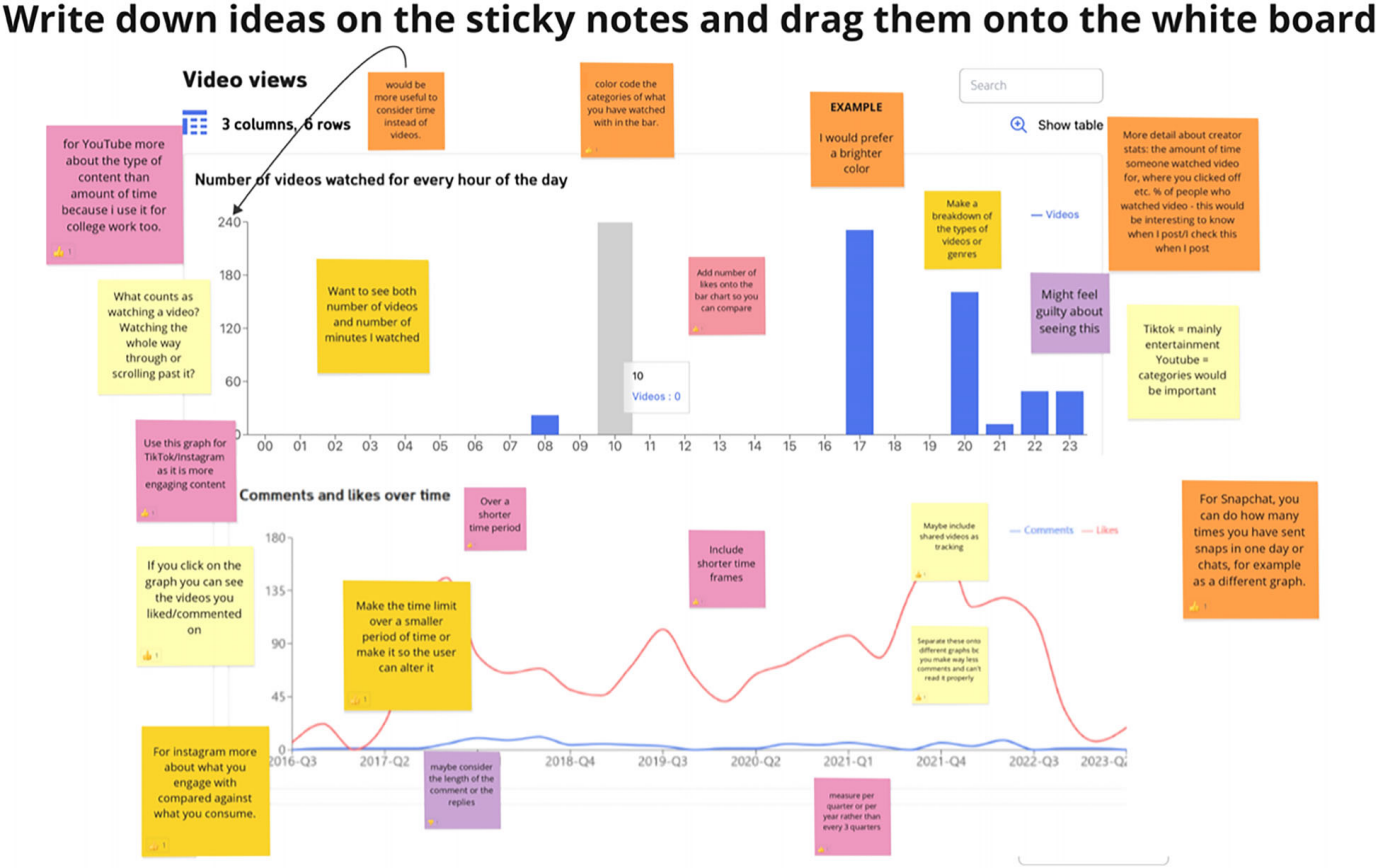
Focus group participants’ (16–18 years old) feedback of the data visualization tool using Miro: To gather participant preferences, we presented an initial mock‐up of the social media data visualizations they would receive after donating their social media data. These visualizations, displayed through graphs, serve as feedback about their data usage. Using the sticky notes in the interactive Miro tool, participants were instructed to provide anonymous feedback on preferred features and potential improvements for refining the tool.

Using the Miro platform (Figure 3), we gathered input on preferences for how data should be presented to improve the data visualization tool for our study.

Focus group adolescents favored metrics that reflected the time spent watching videos rather than simply the number of videos watched. These time‐based metrics likely provide a clearer picture of engagement and investment in content, which they found more meaningful and less overwhelming than counting the volume of consumed media. However, there was a noticeable discomfort or guilt associated with seeing the total number of videos they had watched. Although adolescents are curious about their viewing habits, they may also be sensitive to the implications of high consumption, possibly feeling judged or concerned about their time management.

Younger adolescents (13–15 years old) felt that the tool was too “mathematical” and not sufficiently engaging for their age group. They preferred a more visually appealing interface with animations and pictures. Both younger and older adolescents expressed a strong preference for personalized data visualizations, similar to platforms like Spotify Wrapped [31], where they could see their most‐watched content and interactions.

### 10.6 Effective Study Presentations and Communication Strategies

Before the researchers visited schools to deliver presentations on social media and mental health as well as the study, the youth advisory panels’ adolescents played a crucial role in shaping the content and approach of these presentations and removing any barriers to engagement or comprehension. They emphasized the importance of presenting the study in a way that avoids making participants feel guilty about their social media use, as they felt that they have already received enough of this messaging. Instead, the presentation should tap into the natural curiosity adolescents have about how social media impacts their mental health and well‐being. They also suggested that communication requiring parental consent would be more trusted if delivered through teachers and schools rather than directly from the research team. Furthermore, sign‐up resources would benefit from including videos or visual media to complement long text documents. They also highlighted the need to simplify and clarify certain sections of study documents, with a particular emphasis on anonymity and privacy to address participants’ and parents’ primary concerns.

## 11 Recommendations

**FIGURE 4:**
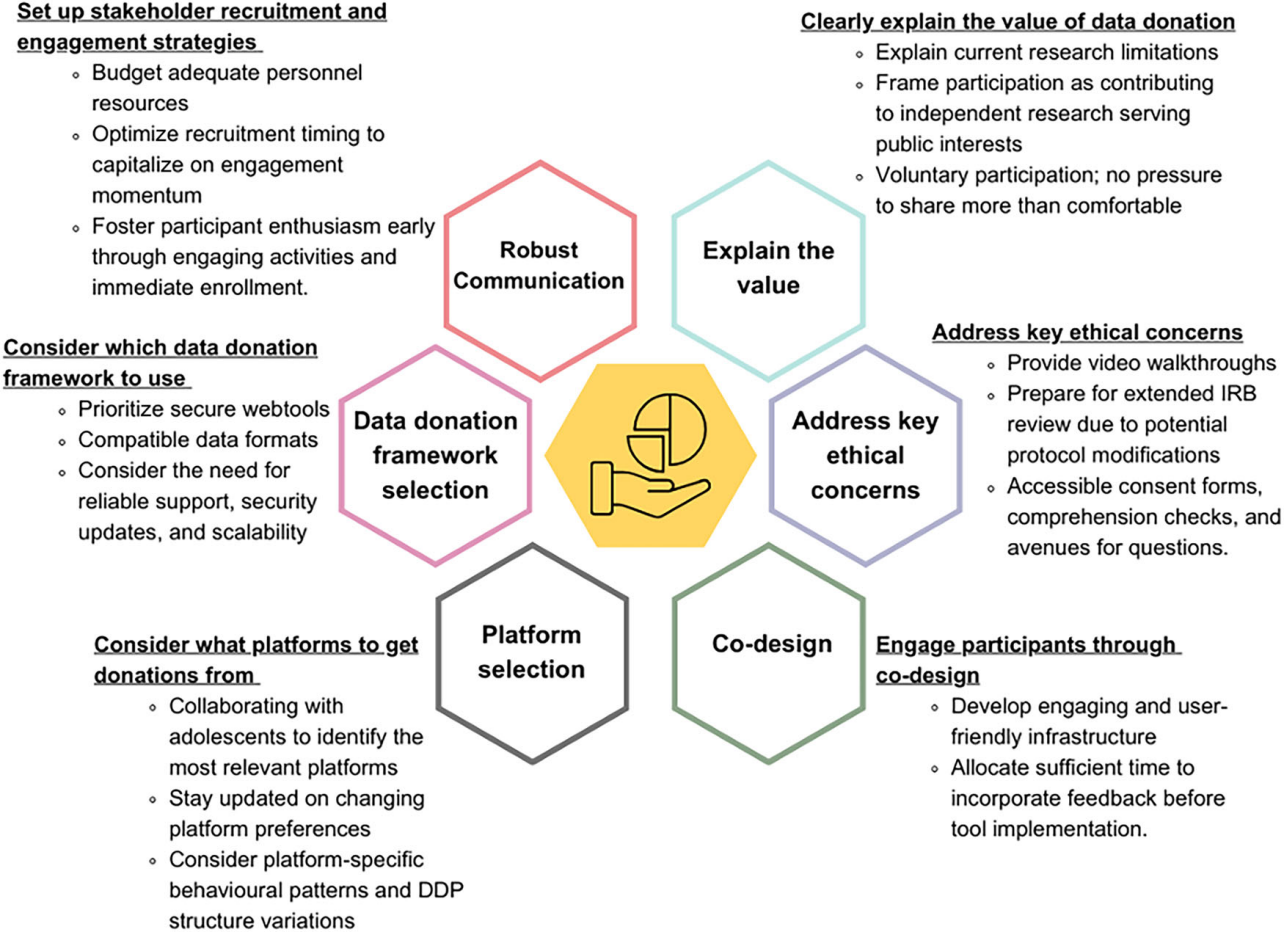
Six key recommendations for effectively engaging adolescents in data donation research.

To aid the design of data donation studies for adolescents in future, we offer six key recommendations for effectively engaging adolescents in such research informed by our experiences (Figure 4). These recommendations aim to amplify the voices and perspectives of adolescent participants, ensuring that future data donation initiatives are effective, supportive, ethically robust, and inclusive.

### 11.1 Recommendation 1: Clearly Explain Value of Data Donation

Data donation requires innovative value communication approaches that move beyond traditional informed consent models. For many adolescents, data donation is a novel concept, and this unfamiliarity, combined with the perceived high effort of participation, can present a significant barrier to engagement unless researchers can demonstrate concrete, personally meaningful benefits that connect to adolescents’ existing digital experiences and concerns.

As an example, we began our school presentations by prompting students to reflect on their own social media use, asking questions like, “What comes to mind when you hear the phrases ‘social media’ and ‘mental health’?” This approach validated adolescents’ existing knowledge and concerns while creating natural transitions into discussing research objectives.

Adolescents demonstrated strong engagement when we explained the current limitations in social media research, including how platforms control access to user data and may have different priorities than independent researchers. We discussed how data donation enables research that is free from commercial influences and platform restrictions, allowing researchers to ask questions that platforms might not prioritize. This transparency about research constraints and the role of corporate data policies helped participants understand how their contributions support independent scientific inquiry that serves public rather than commercial interests.

Moreover, researchers should ensure that participation feels genuinely voluntary. We avoided compensation models tied to the volume or type of data shared, as this could create pressure. Instead, we encouraged participants to share only the data they felt comfortable donating. We also offered vouchers for participation, regardless of data donation. By implementing these strategies, researchers can transform data donation from a transactional process into a collaborative and meaningful engagement, empowering adolescents to contribute to research in ways that align with their values and enhance their sense of agency.

### 11.2 Recommendation 2: Address Key Ethical Concerns

Ensuring that adolescents have a clear understanding of the data donation process and how their data will be used is essential. Unlike impersonal data, DDPs can reveal sensitive information participants may not realize they are disclosing, such as mental health status, political beliefs, or sexuality [32]. Therefore, consent processes need to be extensive, including (1) a short, plain language information sheet; (2) a test of comprehension; (3) a live or video demonstration of a data donation process; and (4) an avenue for participant to ask clarifying questions.

To address concerns about data privacy and participant anonymity, researchers should also be transparent about the measures they take to protect participant data. These measures could include the use of pseudonyms, data encryption, and strict data handling protocols. These procedures should be clearly documented in the consent procedure to reassure about the security of sensitive information. It is important that the information be conveyed in an accessible manner, avoiding superfluous technical details.

Three key aspects should be carefully considered to aid participant consent and data privacy. First, participants typically store their DDPs locally on their devices. After participation in any study, they should be informed about the presence of these packages and given the option to either keep them under their own responsibility or delete them permanently. Second, to enhance transparency and ensure informed consent, participants should be shown examples illustrating what information will be extracted from their DDPs. In our study, we screen‐recorded the entire process of data donation, highlighting what specific data would be visible to researchers. This demonstration was presented during our school recruitment pitch and made publicly available on our website.

Furthermore, researchers need to consult the ethical review boards of their institutions, which, due to the novelty of the method, might require substantial engagement before ethical approval is granted. For example, our participant recruitment range was initially set at 8–18 years, to reflect social media demographics. However, due to the legal age limits of most platforms, which typically require users to be 13 or older, we were advised to narrow the participant age range to those above this threshold to ensure compliance.

A final concern was the potential identifiability of participants through their donated social media data. During the initial planning phase, we considered examining certain elements of the DDPs, such as links to videos liked on TikTok. Although such videos are often publicly available, the act of liking them reflects a personal interaction and, in some cases, particularly if the video has a low number of total likes, could increase the risk of participant re‐identification even if any directly identifiable personal data are removed. Our institutional ethics board flagged this as a concern. In response, we refined our data collection strategy to exclude any elements that could be publicly linked to an individual through triangulation. Specifically, we did not collect private messages or any data intended to be shared privately. Although some videos may incidentally feature other individuals, our analysis was limited to public content voluntarily shared by participants, and we avoided any interpretation of interactions (e.g., comments, likes) involving other users. Participants were also assigned a randomly generated ID to anonymize their data, which facilitated the linking of relevant data aspects while preserving anonymity. These adjustments were essential in upholding participant privacy and maintaining the ethical standards of our research.

### 11.3 Recommendation 3: Engage Participants Through Co‐Design to Enhance Study Experience

Given the high‐effort nature of data donation, it is essential to create an engaging and user‐friendly data donation infrastructure to motivate adolescent participants. A key starting point is to understand the population of interest, such as their age distribution, their proficiency with technology, and their attitudes towards research.

Adolescents must trust not only the researchers but also their own ability to navigate the technical aspects of the process. Prior to the feasibility study, we conducted a pilot study with four participants who went through the entire study flow and tested the data donation tool. They appreciated the data visualizations, viewing them as a valuable trade‐off for their participation. However, they also provided feedback on some technical difficulties they experienced, such as difficulties in locating the DDP files they downloaded or issues with files appearing empty. This allowed us to make necessary adjustments before running the feasibility study.

We found that co‐designing with adolescents was instrumental in creating a tool that resonated with them. If participants struggle with navigating the process or become frustrated, they may unintentionally provide inaccurate data or abandon the process prematurely. For future studies, we recommend conducting several co‐design workshops early in the project to incorporate feedback into the development of the data donation tool. We would also advise allowing sufficient time between the co‐design phase and implementation to fully incorporate feedback.

### 11.4 Recommendation 4: Consider What Platforms to Get Data Donations From

Young people are among the most active users of digital platforms. For example, they typically use nine different social media sites regularly, compared to six for the average adult user [33]. In recent years, Snapchat and TikTok have become the most popular platforms among 16‐ to 24‐year‐olds, surpassing Instagram. BeReal, an app that encourages users to share a daily, unfiltered photo, has also gained traction, with usage growing from 9% to 22% between spring and autumn 2022 [28]. It is beneficial to collaborate with adolescents, such as through focus groups or advisory panels, to determine which platform data is most relevant to collect for research. Collecting screen time data in a pre‐survey can also help confirm which platforms are important to study. The popularity of social media apps can shift quickly. Researchers need to keep pace with these changes to target the most relevant data sources, which will allow accurate reflection of the online behaviors and preferences of adolescents.

Beyond popularity metrics, selection of which platforms to request data donations from should consider data richness, DDP quality, and alignment with research questions. Different platforms capture distinct behavioral patterns. For example, Instagram emphasizes visual content and social comparison, and TikTok reveals content consumption and algorithmic exposure, while messaging platforms like Snapchat provide insights into private communication patterns. However, DDP quality can vary considerably; for instance, Valkenburg et al. [34] noted that DDP structure varies significantly in data completeness and consistency, which limits research utility. Combined with differences in temporal coverage and analytical structure across platforms, these factors require researchers to weigh the available data elements carefully against their study objectives.

### 11.5 Recommendation 5: Consider Which Data Donation Framework to Use

Data donation frameworks are the software systems and tools that researchers use to collect, process, and manage donated data. Framework selection directly impacts study success. Given the variety of open‐source data donation frameworks available, it is important to choose a framework that balances security, ease of use, and long‐term sustainability—this might vary across different projects. One might also decide to build and host data donation infrastructure individually, yet this can be challenging.

Server‐side processing frameworks, exemplified by the Data Donation Module [39], centralize data handling on researcher‐controlled infrastructure, which enables sophisticated analytical capabilities and standardized processing workflows. However, this approach requires participants to transmit complete DDPs to external servers, potentially raising privacy concerns and institutional review board complications, particularly when handling sensitive adolescent data. Conversely, client‐side processing frameworks such as the Port webtool [40] and components of the Open‐Source Data Donation Framework (OSD2F) [41] perform initial data filtering and aggregation locally within participants’ web browsers before any data transmission occurs, reducing the sensitivity and scope of the data collected and minimizing the risk of unnecessary disclosure.

However, client‐side processing introduces significant technical complexity, requires sophisticated JavaScript/Python integration (often through technologies like Pyodide), and limits analytical capabilities to computations feasible within browser environments. The debugging and quality assurance challenges for client‐side processing are substantially greater, as researchers must account for diverse browser configurations, security settings, and participant technical literacy levels. For more comprehensive information about the technical setup of data donation frameworks, please see Carrière et al. [42].

Framework sustainability encompasses active developer participation, institutional backing, and long‐term maintenance beyond technical functionality. Although open‐source frameworks offer customizability and transparency, they depend on volunteer contributions and lack guaranteed maintenance, whereas academic‐led projects face funding cycle vulnerabilities [43]. Emerging institutional services like the Smart Data Donation Service (SDDS) represent a different sustainability model through establishing a national research infrastructure that recruits data donor cohorts and provides researchers access to centralized repositories rather than requiring independent framework implementation.

Strategic framework selection requires balancing technical capabilities with practical constraints and sustainability considerations. Researchers should evaluate frameworks against specific criteria: data processing approach, privacy protection mechanisms, technical expertise requirements, integration capabilities, and long‐term sustainability indicators. This systematic assessment ensures alignment between framework capabilities and research objectives while avoiding costly mid‐study platform transitions.

### 11.6 Recommendation 6: Set Up Comprehensive Multi‐Stakeholder Recruitment and Engagement Strategies

Executing a data donation study, especially one involving adolescents, is a complex and resource‐intensive endeavor that requires extensive planning, communication, and expertise.

To reach a diverse range of participants, our team targeted different towns and cities across the United Kingdom. We emailed psychology and social science teachers, followed by phone calls to explain the project and its benefits and to schedule in‐person visits. To more effectively engage students and provide reciprocal value to the schools, two members of the research team visited seven schools to deliver an interactive, 1‐h presentation. The session not only introduced the research but also included engaging tasks (e.g., using post‐it notes to write down research ideas). In line with feedback from our youth advisory panels, we carefully framed the discussion to avoid inducing guilt about screen time or portraying social media negatively. Recognizing that not all students or parents could attend the in‐person session, we organized weekly Zoom drop‐in sessions to present the study opportunity.

As we conducted the study on a rolling basis, we observed that participants who enrolled shortly after our school visits were more likely to engage fully with each part of the study, demonstrating higher levels of enthusiasm and a lower dropout rate. The in‐person presentations played a crucial role in sustaining participant interest and commitment. Younger students (aged 13–15 years) had the lowest sign‐up rates, likely due to the additional effort required to obtain parental consent, which involved parents checking their emails and responding. The need for parental involvement created a barrier to participation; therefore, streamlined consent processes and the need for more direct communication channels with parents to facilitate timely responses will be important in future to boost retention.

Researchers should develop stakeholder‐specific communication strategies that acknowledge distinct motivations, concerns, and decision‐making processes. School engagement requires emphasizing educational reciprocity and curriculum alignment, whereas adolescent recruitment benefits from interactive, nonjudgmental approaches that validate participants’ digital experiences. Parental engagement also demands transparent, accessible communication about research benefits and robust privacy protections. Future studies should budget accordingly for sustained personnel support rather than treating recruitment as a brief initial phase.

## 12 Discussion and Conclusion

Data donation represents a substantial opportunity to improve how we understand adolescent online behaviors, especially at a time when conventional data collection methods cannot collect information that parallels the intricacies of adolescents’ online experiences. Our feasibility study, which successfully engaged over 300 adolescents and collected 275 DDPs from TikTok (65.8% donation rate) and Instagram (78.9% donation rate), demonstrated that large‐scale adolescent data donation is both feasible and well‐received.

Further, it has led us to propose a series of adolescent‐centered recommendations for future data donation studies with young people. Researchers need to ensure they are (1) clearly explaining the value of data donation, (2) establishing ethical consent procedures, (3) engaging participants through co‐design to enhance the study experience, (4) selecting appropriate digital platforms to get data donations from, (5) choosing the right software and frameworks for implementing the data donating process, and (6) setting up comprehensive multi‐stakeholder recruitment and engagement strategies.

Our research revealed that many adolescents, especially younger participants, initially found the concept of data donation confusing and expressed concerns about sharing sensitive information. Clear communication, informed by adolescent advisors and focus groups, was crucial to clarify the process and make it accessible. Highlighting the value of data donation was key: Once adolescents understood how their contributions could improve research, they felt more motivated and empowered to participate. By ensuring transparency around data‐handling practices and creating reciprocal value through co‐design, we also built trust and sustained participant engagement.

However, it is important to recognize that data donation is no panacea. Conducting a data donation study comes with its own set of challenges, primarily related to financial costs and technological unpredictability. Establishing a secure and user‐friendly platform for data collection often requires substantial investment, especially when integrating new tools. Platforms like the Port webtool are addressing this issue by developing community resources and open‐source materials that support independent infrastructure setup [30]. This shift aims to reduce costs associated with external hosting services, making it more feasible for research teams; however, it still exceeds many research budgets. Researchers also face the unpredictability of technology companies, as social media platforms frequently change their data formats or policies regarding data access. These changes can disrupt the data donation process, complicating the collection of DDPs and necessitating ongoing adjustments to research protocols.

However, data donation and the rich data it provides open many new potential research pathways. Researchers are now, for example, beginning to explore the potential of combining participant‐donated social media data with both human annotation and computational tools. For instance, recent studies have used data donation workflows to collect adolescents’ social media posts—texts, images, and story data—which were then manually coded to capture emotional expression and linked to well‐being measures [44, 45]. In parallel, advances in natural language processing (NLP), computer vision, and large language models (LLMs) have opened new avenues for analyzing DDPs at scale. Recent work demonstrates how LLMs can be fine‐tuned for psychological constructs such as self‐doubt or rumination by adding classification layers to models trained on curated, theory‐driven datasets [46, 47]. Instruction‐tuned models can also be prompted for classification tasks, though this requires careful engineering and validation.

At the same time, growing interest in data donation highlights the need to center adolescents’ perspectives. It is vital to recognize that adolescents are independent actors and rights‐holders in the digital environment [48]. Data donation, in this context, empowers adolescents by giving them a role in understanding how social media and other digital platforms impact their lives. It allows them to gain insights into the data social media companies collect from them and promotes a more informed and reflective approach to their own usage. As digital technologies continue reshaping adolescent development, research methodologies must evolve to match both the complexity of these environments and the sophistication of the young people navigating them. It is critical that we remain responsive to the voices, perspectives, and concerns of adolescents, ensuring that data donation is used to enhance their digital well‐being and empower them in their digital environments.

## Author Contributions

**Valerie Z. Y. Yap**: writing – original draft preparation, data curation, methodology, project administration, investigation, visualization. **Amira Skeggs**: writing – review and editing, methodology, investigation. **Amanda M. Ferguson**: conceptualization, writing – review and editing, methodology, project administration. **Amelia Leyland‐Craggs**: methodology, investigation, project administration. **Laura Boeschoten**: software, writing – review and editing. **Kasper Welbers**: software, writing – review and editing. **Sebastian Kurten**: funding acquisition, conceptualization, project administration, writing – review and editing, supervision. **Amy Orben**: funding acquisition, conceptualization, supervision, writing – review and editing.

## Conflicts of Interest

In the past 36 months, A.O. has received funding from the Jacobs Foundation, UK Research and Innovation (incl. the Medical Research Council, the Economic and Social Research Council, and the Engineering and Physical Sciences Research Council), the UK Department for Science, Innovation and Technology, the National Institute of Health, the University of Cambridge, Emmanuel College of the University of Cambridge, and the Livelihood Impact Fund. She was an unpaid member of the ESRC Smart Data Research UK Programme Board, British Academy Public Policy Committee, UK Department for Education Science Advisory Council, UK Department for Science, Innovation and Technology and UK Department for Culture, Media and Sport College of Experts, Australian eSafety Commissioner Social Media Minimum Age Evaluation Academic Advisory Group, and a paid member of the Digital Futures for Children Centre Advisory Board. She has received payment for lectures from SWGfL and Apple University; she also received consulting fees from Innovate UK through Opalescent LTD.

## Supporting information




**Supplementary Material**: nyas70140‐sup‐0001‐SuppMat.docx
