# Supplementary material for: Digital Data Donation With Adolescents
Source: Ann N Y Acad Sci. 2025 Nov 10;1554(1):251–66. doi: 10.1111/nyas.70140 (PMC12728328; doi:10.1111/nyas.70140)

**Supplementary Information**

Table of Contents

[**Figure 1.** *Data donation collection overview.* 1](#_Toc201329064)

[TikTok Metrics Extracted from DDP 2](#_Toc201329065)

[**Figure 2.** *Engagement patterns by posting hour on TikTok.* 2](#_Toc201329066)

[**Figure 3.** *Temporal patterns of user engagement on TikTok.* 2](#_Toc201329067)

[**Figure 4.** *Hourly distribution of video consumption on TikTok.* 3](#_Toc201329068)

[**Figure 5.** *Time Spent on TikTok.* 4](#_Toc201329069)

[**Figure 6.** *Direct message activity on TikTok.* 4](#_Toc201329070)

[Instagram Metrics Extracted from DDP 5](#_Toc201329071)

[**Figure 7.** *Instagram posting activity.* 5](#_Toc201329072)

[**Figure 8.** *Instagram engagement activity.* 6](#_Toc201329073)

[**Figure 9.** *Instagram content consumption.* 7](#_Toc201329074)

[**Figure 10.** *Time spent on Instagram.* 8](#_Toc201329075)

[**Figure 11.** *Number of direct messages sent* 8](#_Toc201329076)

As of May 20, 2024, our data collection phase concluded with a total of 124 TikTok and 150 Instagram DDPs. Participants were able to visualise their social media metrics on Next, using a unique link. Figures 2–11 present data visualisations based on mock Instagram and TikTok DDPs. Well-designed visualisations can help participants explore the data they are donating and make the data donation process more engaging and educational.

### **Figure 1.** *Data donation collection overview.*

**
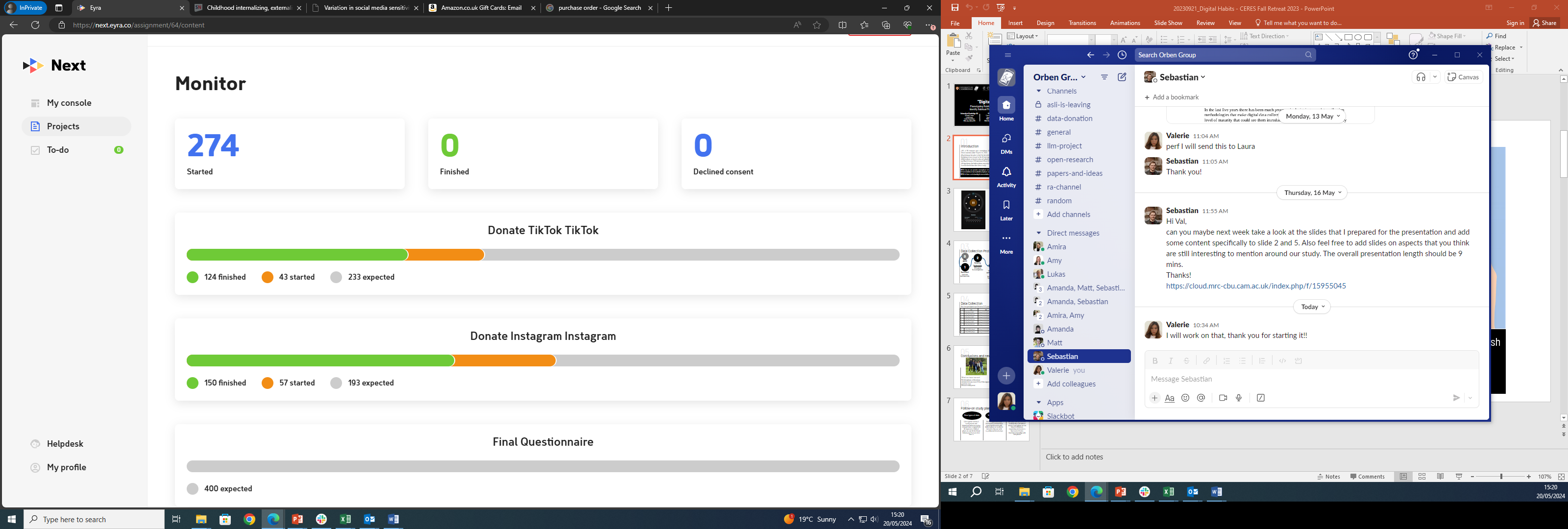
**

## **TikTok Metrics Extracted from DDP**

### **Figure 2.** *Engagement patterns by posting hour on TikTok.*

The table displays the number of videos posted and total likes received, grouped by the hour of posting


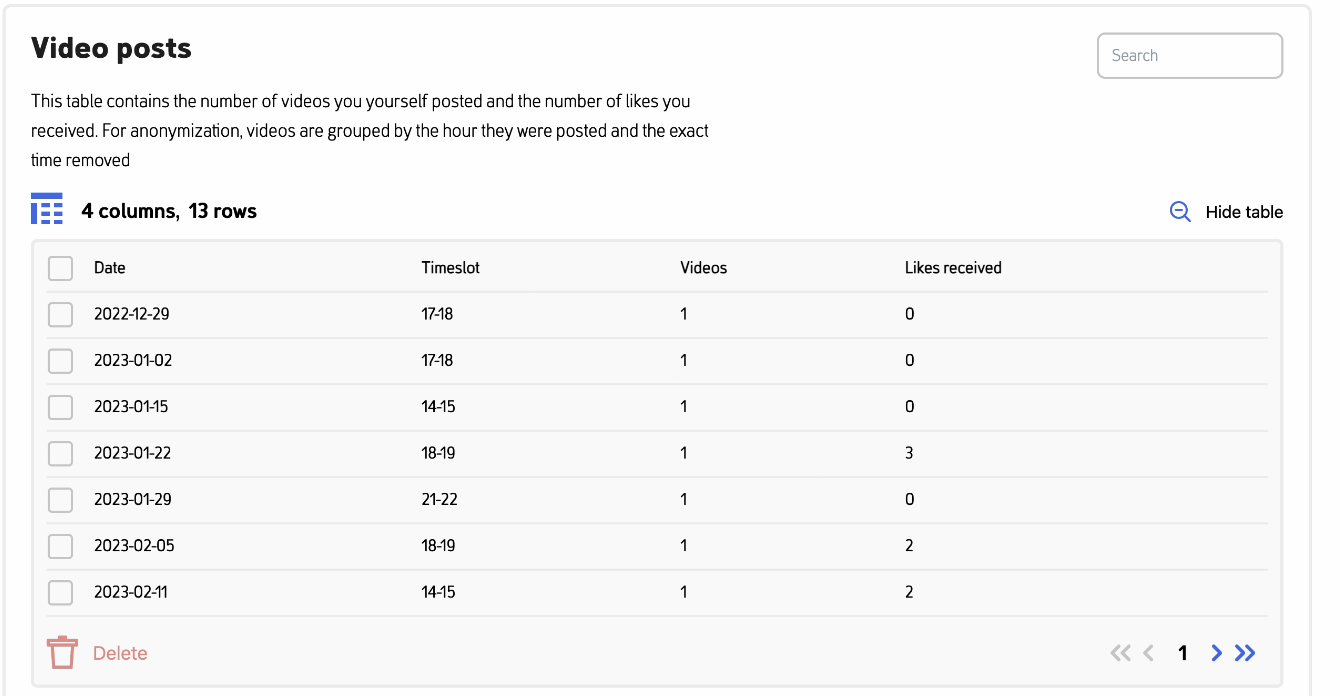


### **Figure 3.** *Temporal patterns of user engagement on TikTok.*

This figure shows the number of likes and comments given, grouped by the hour they were posted.


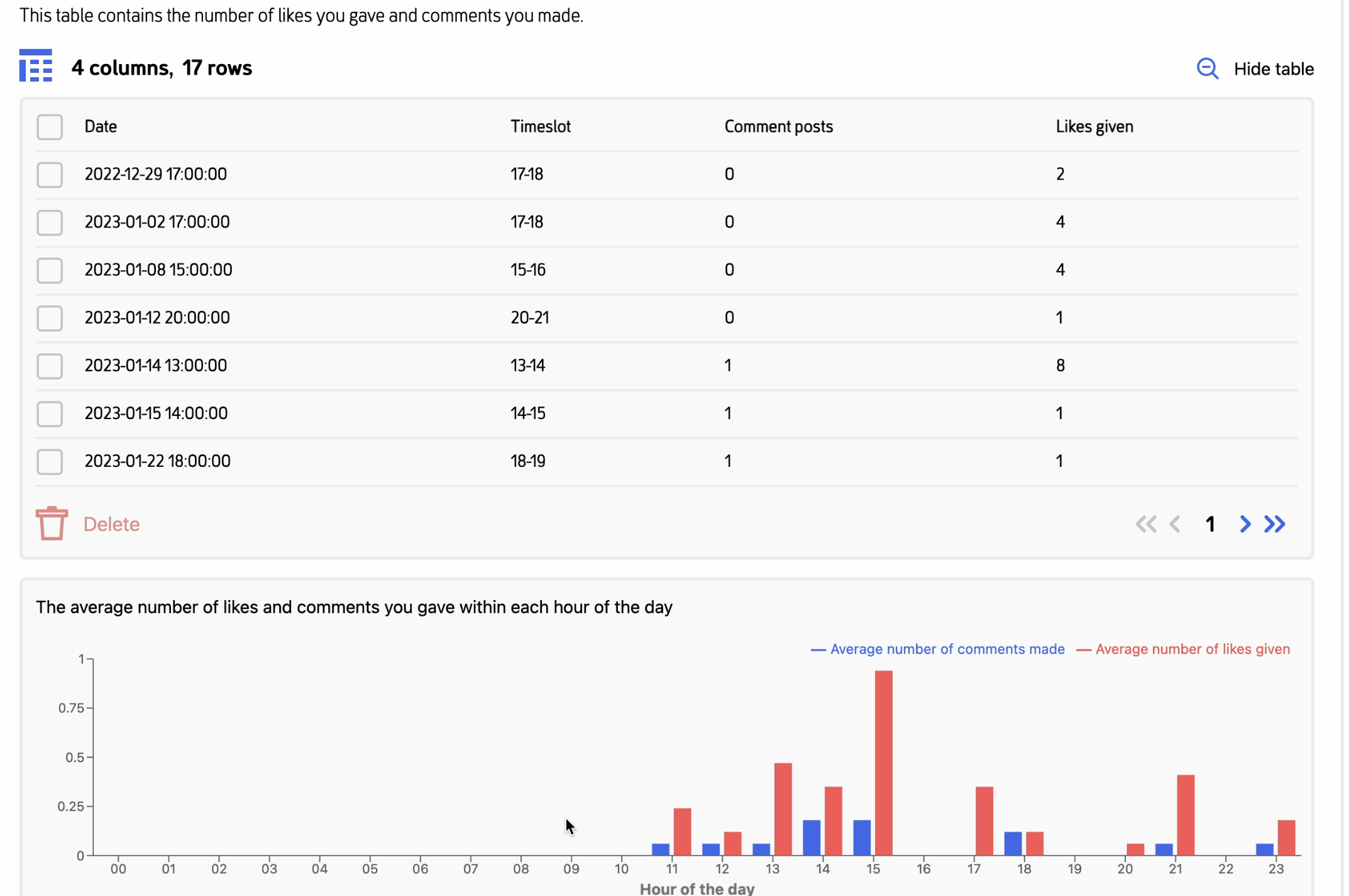


### **Figure 4.** *Hourly distribution of video consumption on TikTok.*

This figure displays the number of videos watched (with associated links) grouped by hour, calculated as a percentage of the total daily videos viewed.


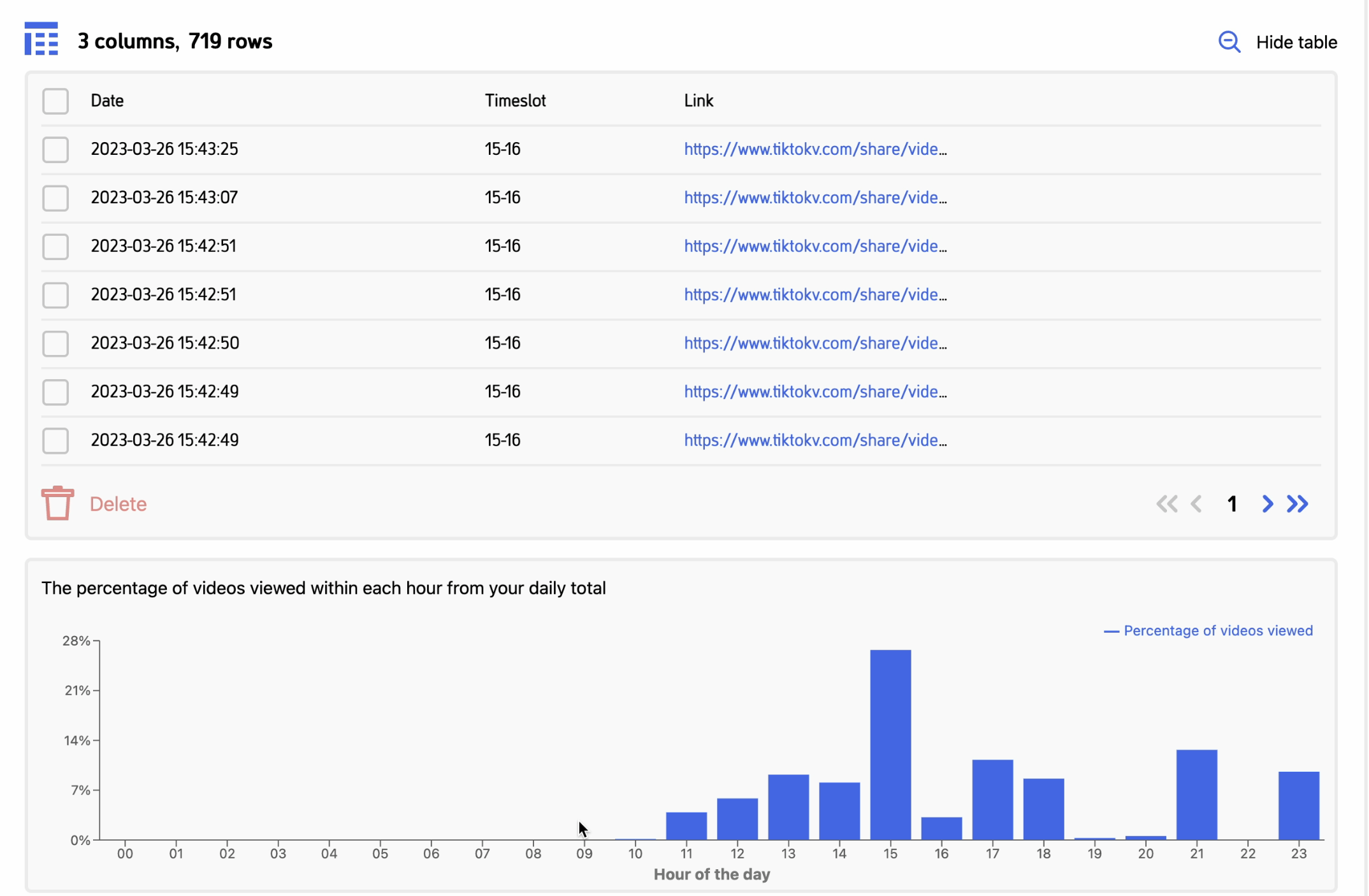


### **Figure 5.** *Time Spent on TikTok.*


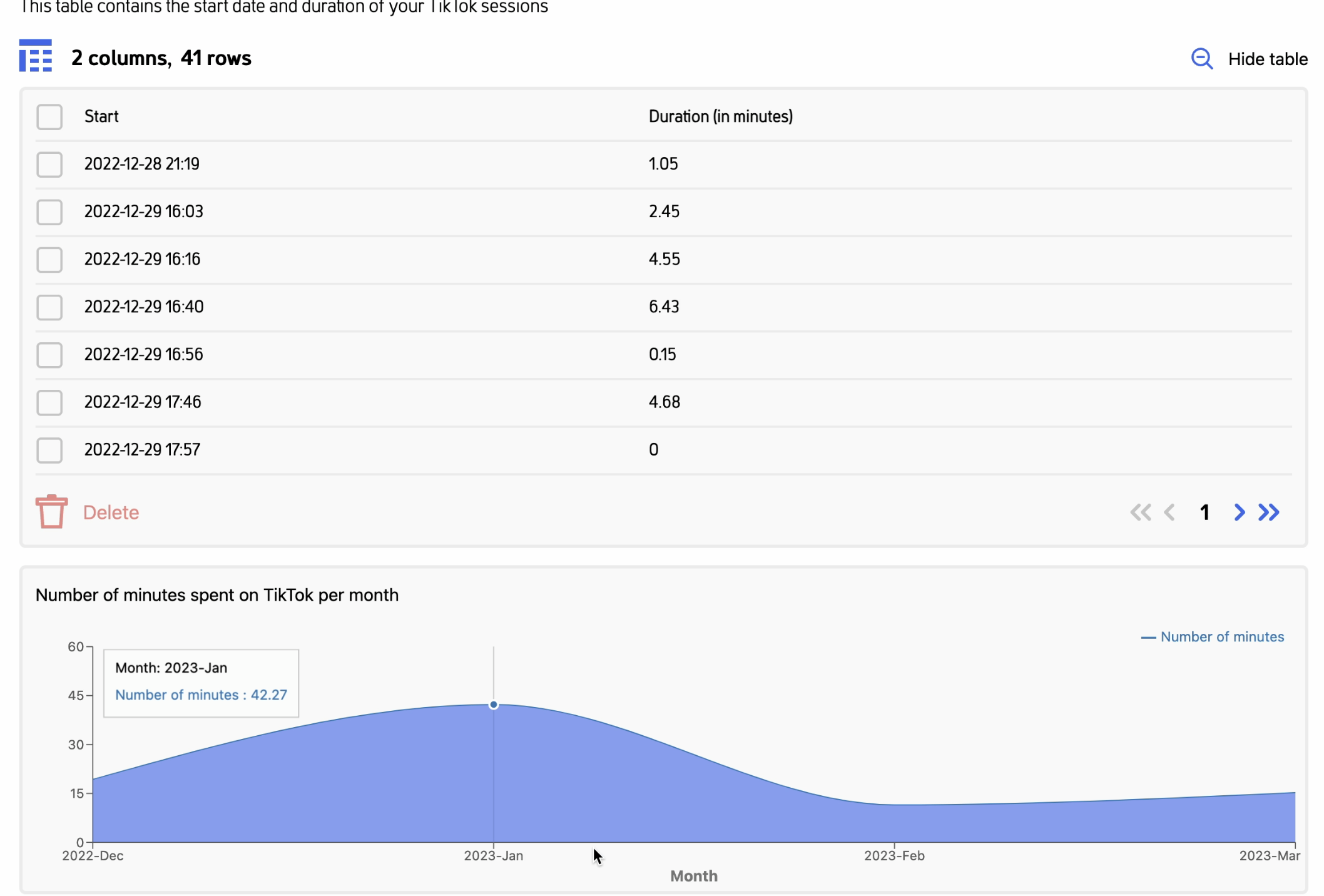


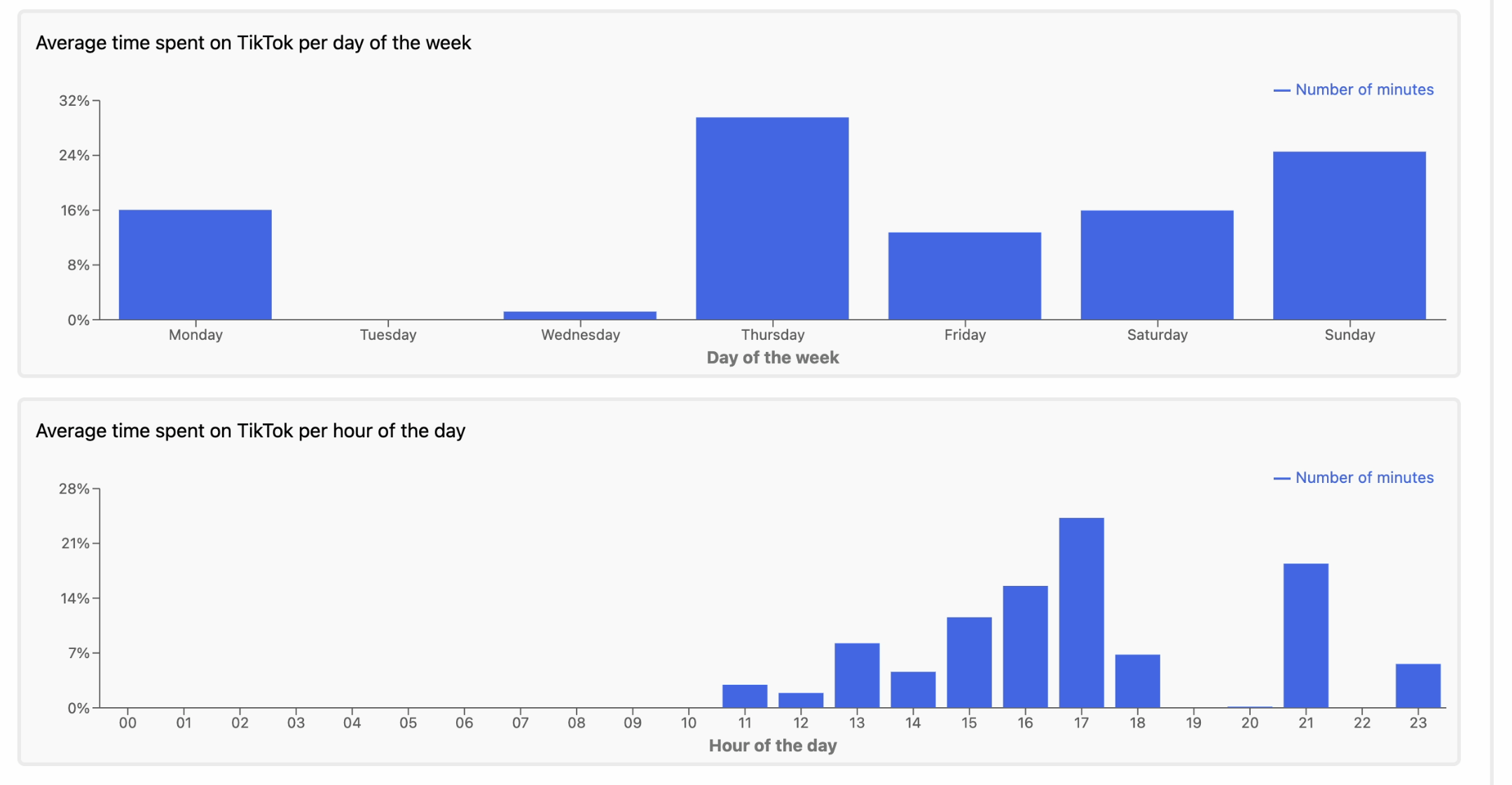


### **Figure 6.** *Direct message activity on TikTok.*

Total number of messages sent and received, grouped by time of day.


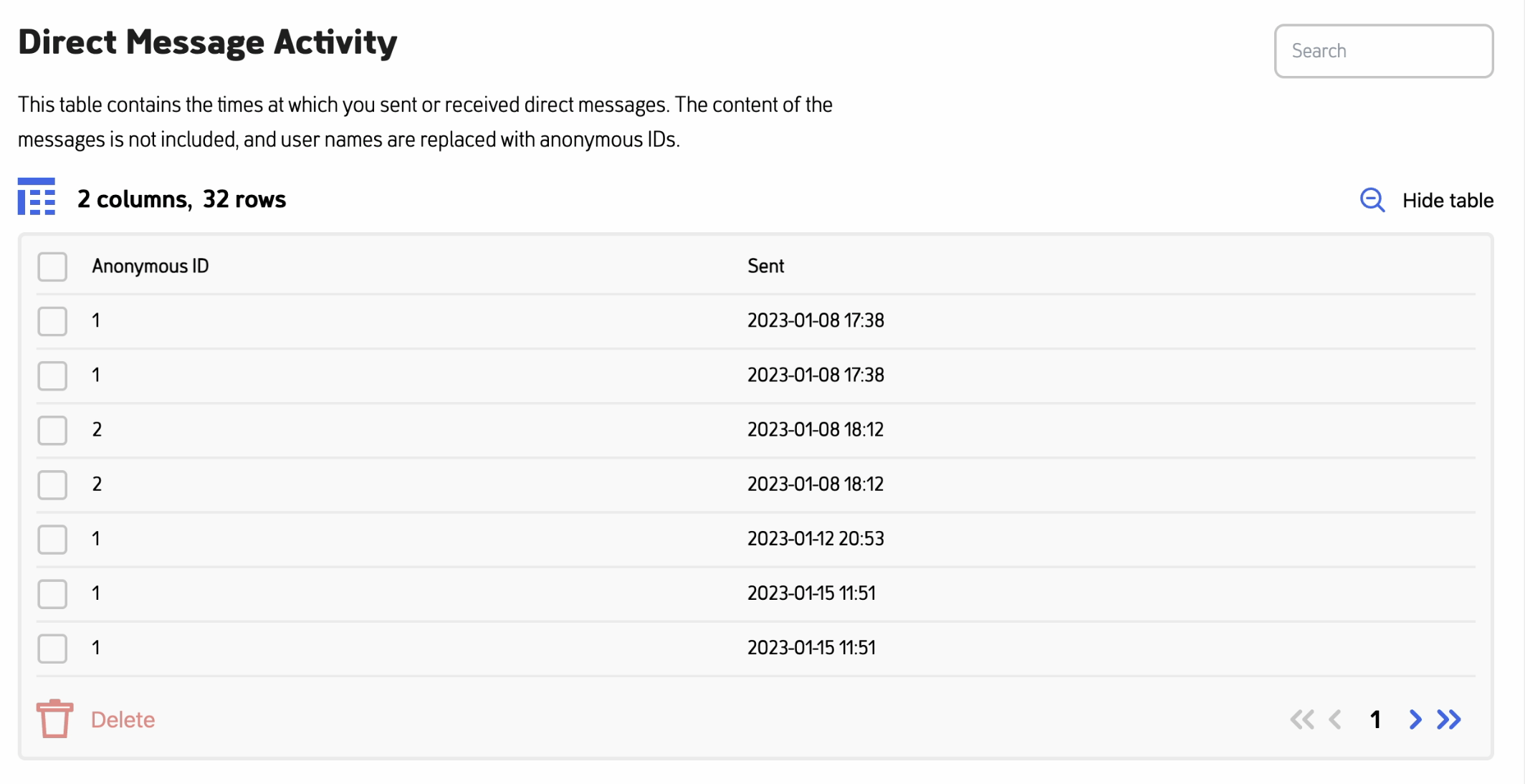


## **Instagram Metrics Extracted from DDP**

### **Figure 7.** *Instagram posting activity.*

Number of feed and story posts: (a) grouped by hour of posting, (b) total posts per month, and (c) average posts per hour of the day.

**
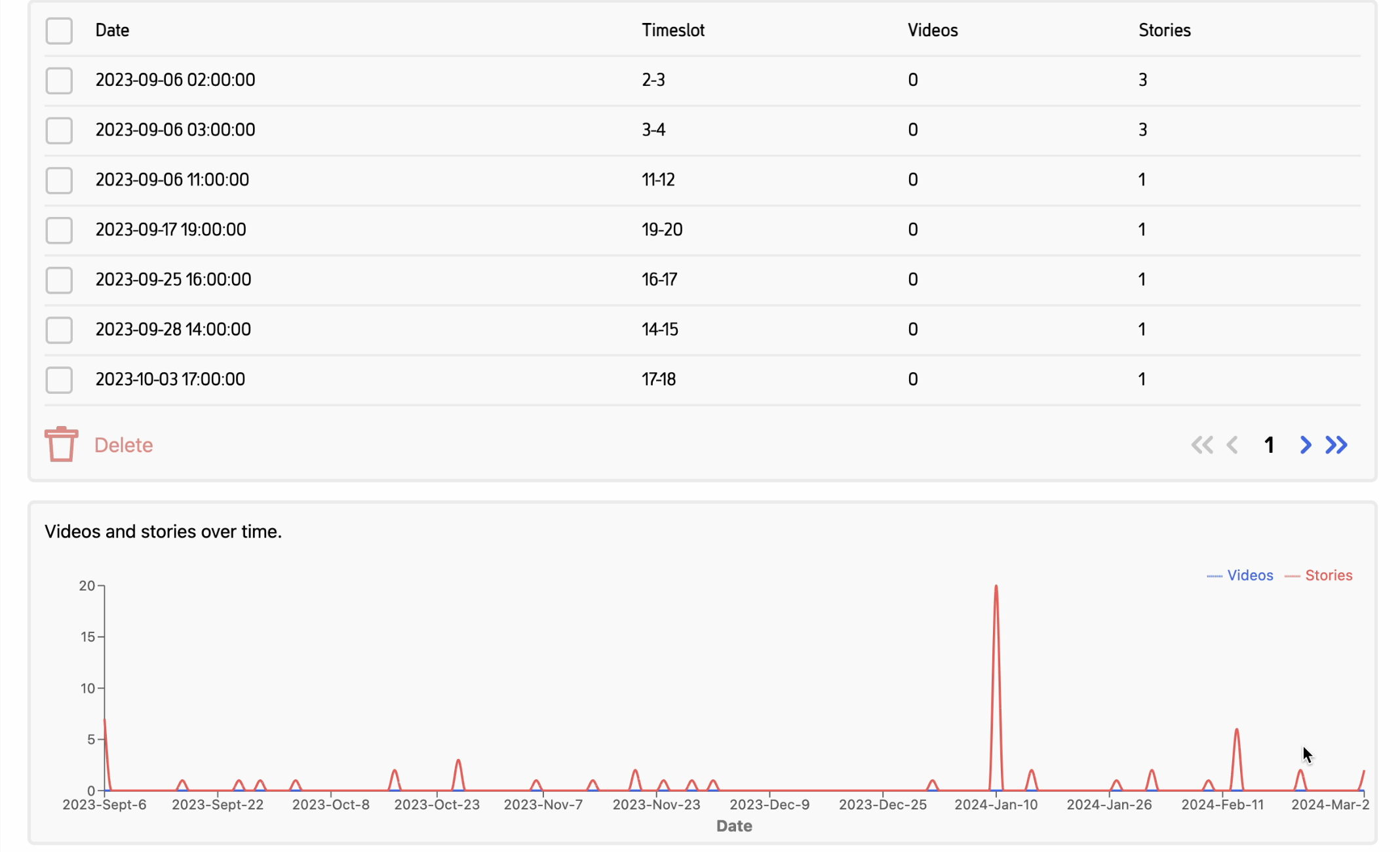
**


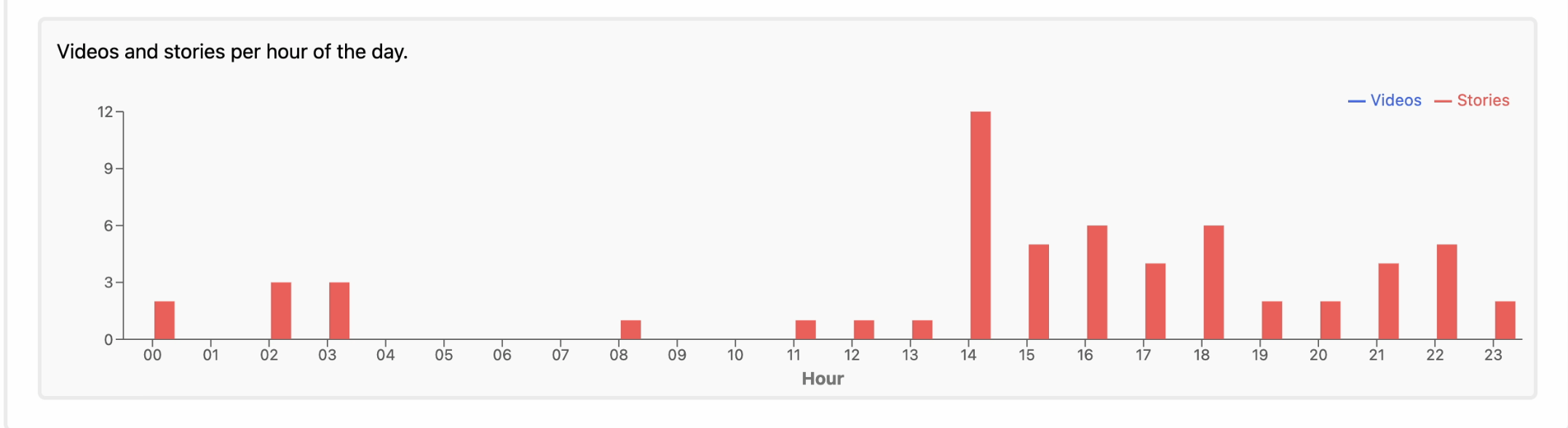


### **Figure 8.** *Instagram engagement activity.*

Number of likes and comments placed by participants: (a) grouped by hour, (b) total per month, and (c) average per hour of the day.


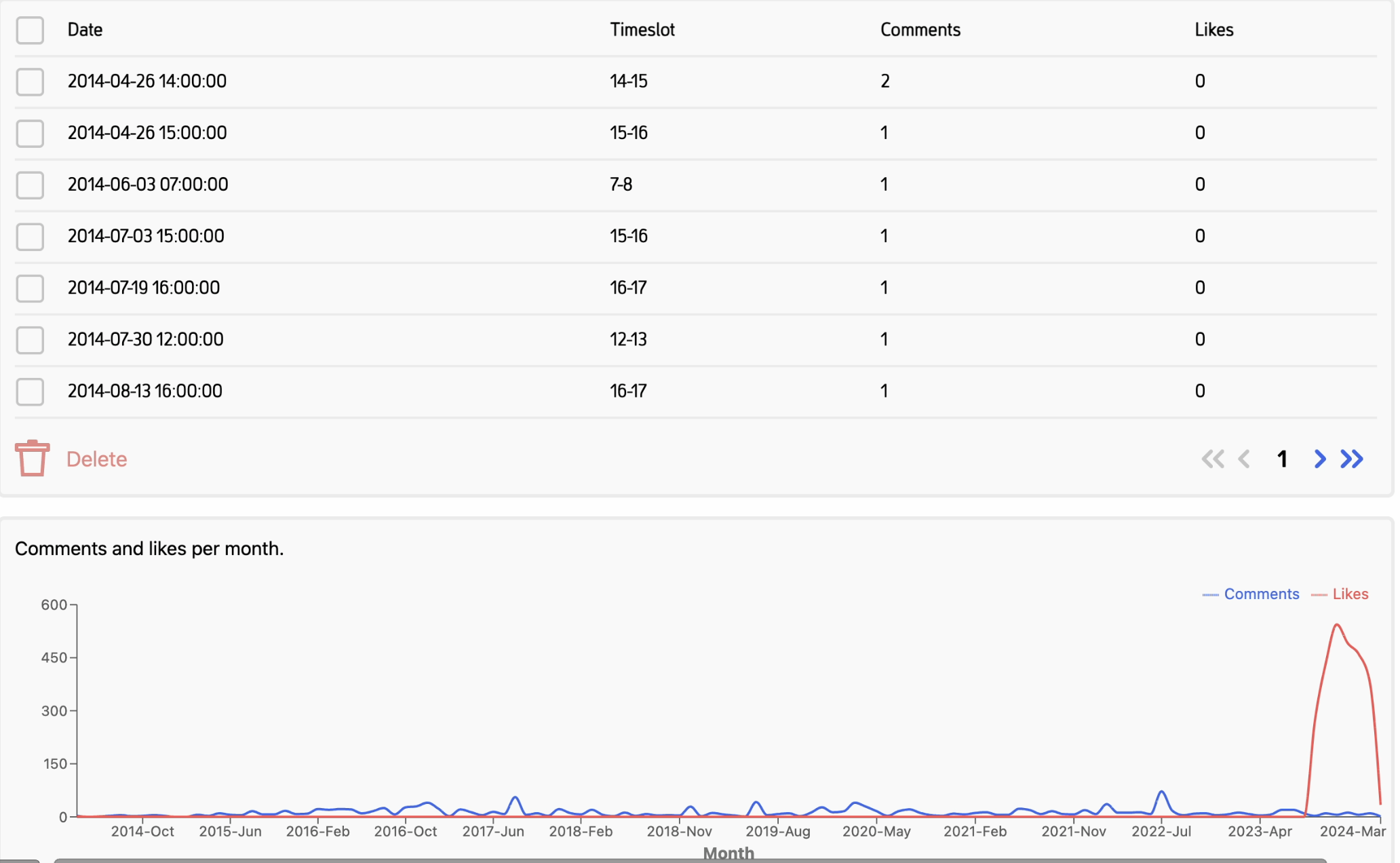


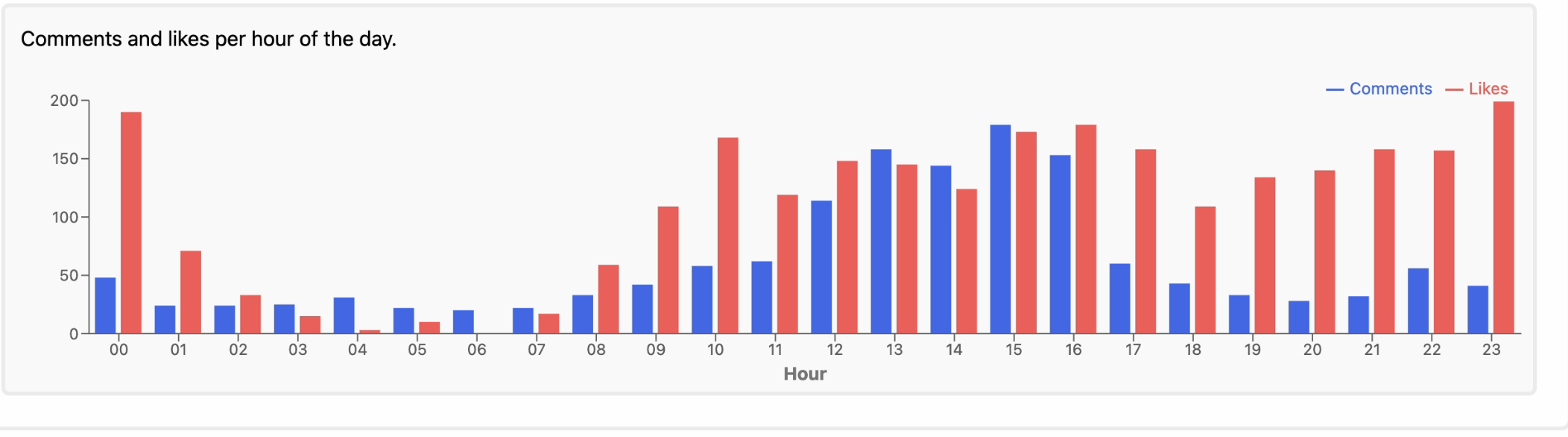


### **Figure 9.** *Instagram content consumption.*

Number of videos and posts viewed by participants: (a) grouped by hour, (b) total per month, and (c) average per hour of the day.


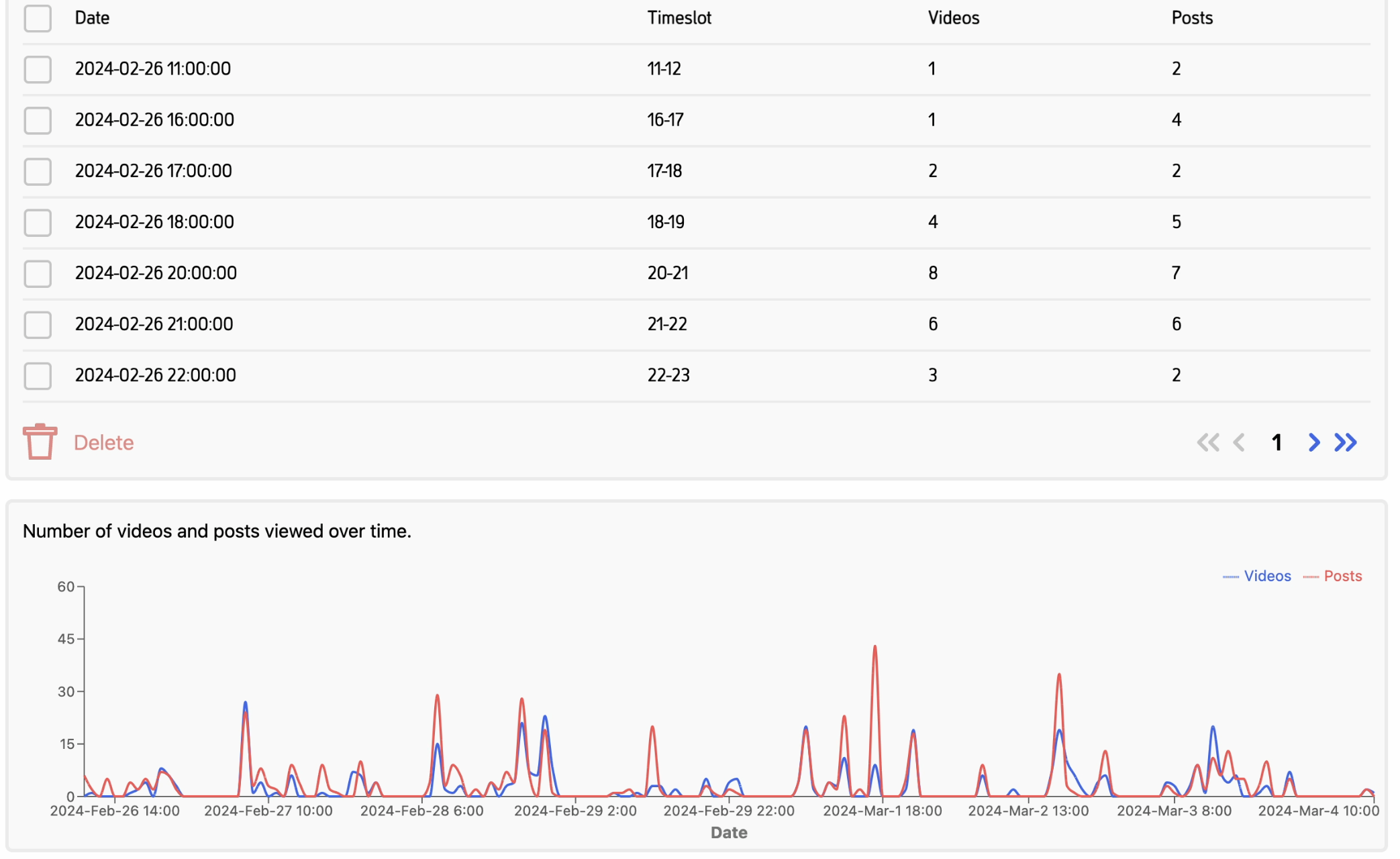


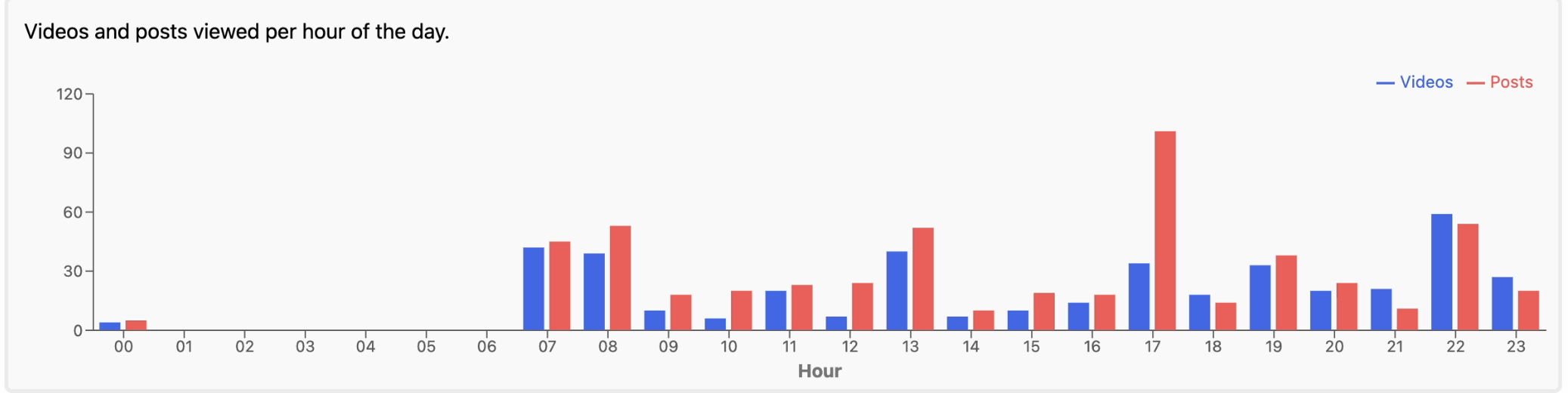


### **Figure 10.** *Time spent on Instagram.*


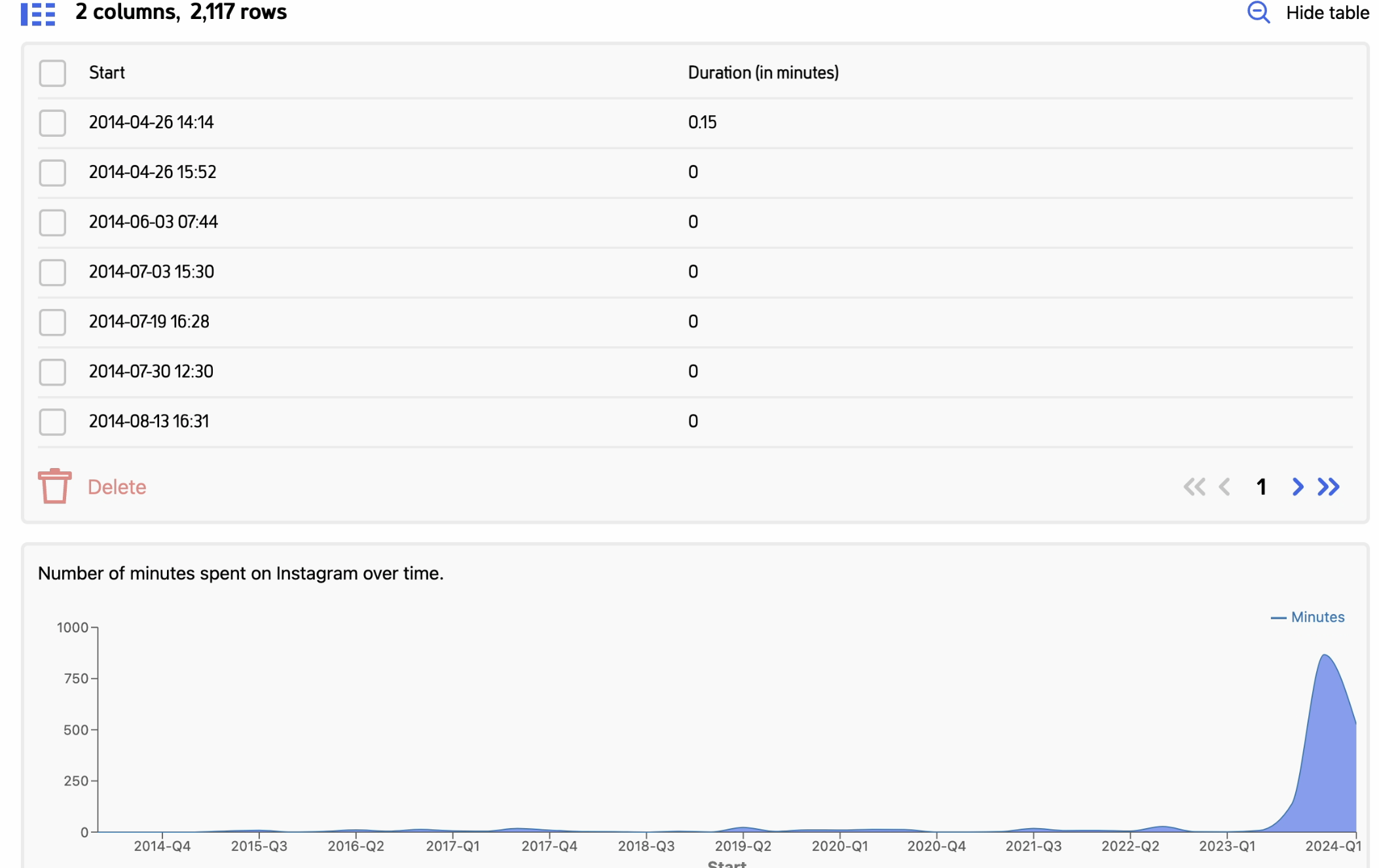


### **Figure 11.** *Number of direct messages sent*


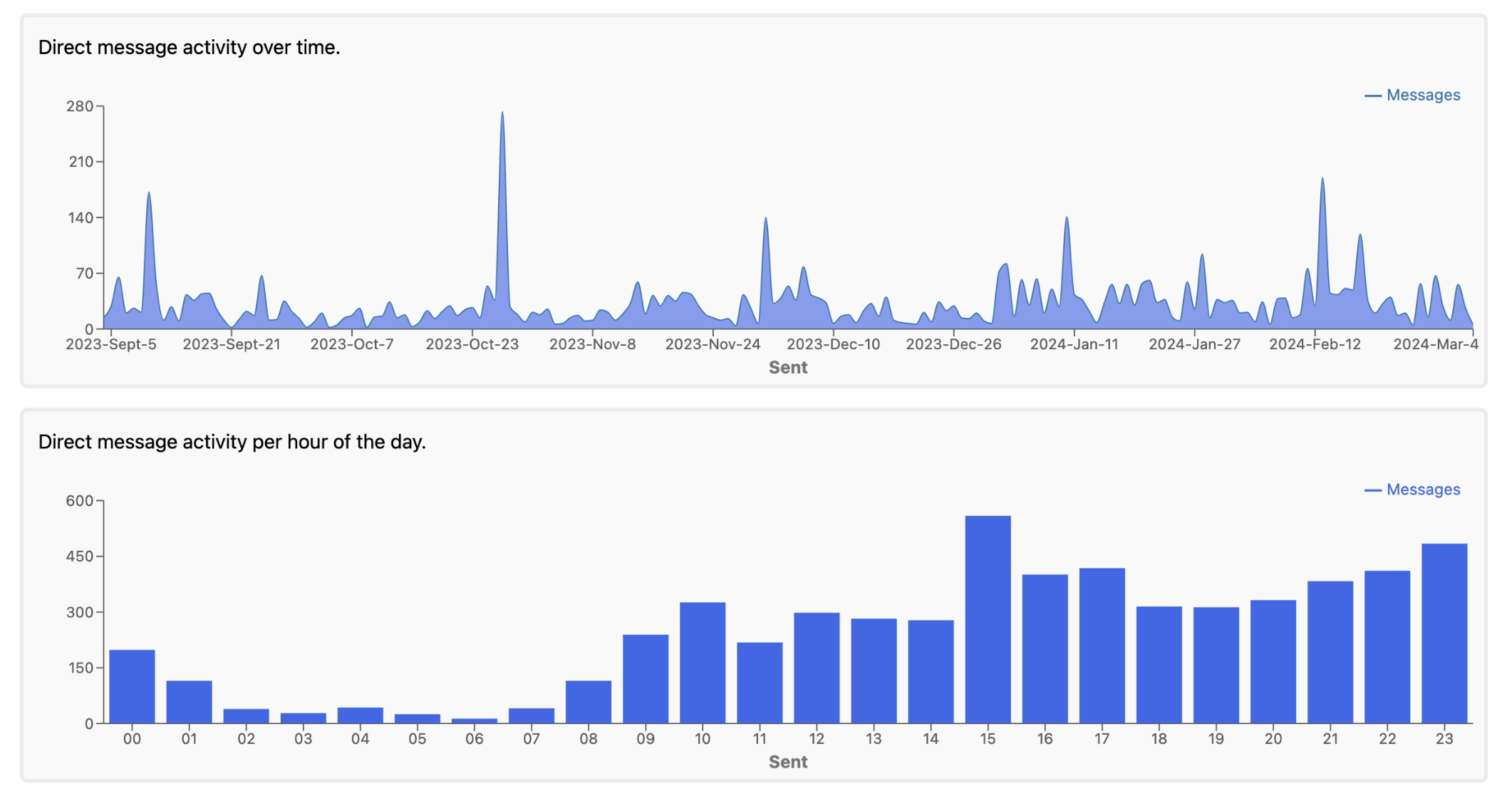

Supplement: Supplementary file 1 — Supplementary Material: nyas70140‐sup‐0001‐SuppMat.docx [file NYAS-1554-251-s001.docx]
